# How Can Ten Fingers Shape a Pot? Evidence for Equivalent Function in Culturally Distinct Motor Skills 

**DOI:** 10.1371/journal.pone.0081614

**Published:** 2013-11-27

**Authors:** Enora Gandon, Reinoud J. Bootsma, John A. Endler, Leore Grosman

**Affiliations:** 1 Computerized Archaeology Laboratory, Institute of Archaeology, Jerusalem, Israel; 2 Institut des Sciences du Mouvement, Aix-Marseille Université, CNRS, Marseille, France; 3 Center for Integrative Ecology, School of Life & Environmental Sciences, Deakin University, Waurn Ponds, Victoria, Australia; Durham University, United Kingdom

## Abstract

Behavioural variability is likely to emerge when a particular task is performed in different cultural settings, assuming that part of human motor behaviour is influenced by culture. In analysing motor behaviour it is useful to distinguish how the action is performed from the result achieved. Does cultural environment lead to specific cultural motor skills? Are there differences between cultures both in the skills themselves and in the corresponding outcomes? Here we analyse the skill of pottery wheel-throwing in French and Indian cultural environments. Our specific goal was to examine the ability of expert potters from distinct cultural settings to reproduce a common model shape (a sphere). The operational aspects of motor performance were captured through the analysis of the hand positions used by the potters during the fashioning process. In parallel, the outcomes were captured by the geometrical characteristics of the vessels produced. As expected, results revealed a cultural influence on the operational aspects of the potters’ motor skill. Yet, the marked cultural differences in hand positions used did not give rise to noticeable differences in the shapes of the vessels produced. Hence, for the simple model form studied, the culturally-specific motor traditions of the French and Indian potters gave rise to an equivalent outcome, that is shape uniformity. Further work is needed to test whether such equivalence is also observed in more complex ceramic shapes.

## Introduction

Human behavioural variability relies strongly on cultural settings that provide specific niches of development [1-4]. This is particularly obvious in motor behaviour. Ethnological studies have documented that the way people sit, swim, walk, carry loads, and so on, is part of the cultural way of life that characterizes each society [5-9]. 

Motor skills result from the interplay of three factors that together define the perceptual-motor workspace: the agent, the task, and the (physical) environment [10,11]. To achieve a particular task, the agent interacts with his/her environment through informational and mechanical couplings [12-15]. Constrained by these factors, the agent’s neuromuscular system is harnessed into functional task-appropriate behaviour. Given the flexibility of the agent’s neuromuscular system a specific task can be achieved by several equivalent motor skills [16]. The set of motor skills of a given task is called the motor space. In the course of motor learning, the individual explores the perceptual-motor workspace in order to discover the perceptual information best suited to accomplish the task at hand [17-19]. Extending this framework to include the social environment, several studies have shown that particular socio-cultural constraints—distinct in individuals from different groups mastering the same task—also channel the learner’s attention [20-26]. This social channel—which corresponds to the cultural transmission of motor skill [27]—not only facilitates the learning process but also paves the way to the development of a specific, culturally situated, motor skill [28]. Thus, the artisan’s exploratory activity occurs over an optimal area of the workspace comprising both the motor space and the social channel. Given that human motor behaviour is influenced by culture, behavioural variation among populations is likely to appear when a particular task is performed in different cultural settings. 

**Figure 1:**
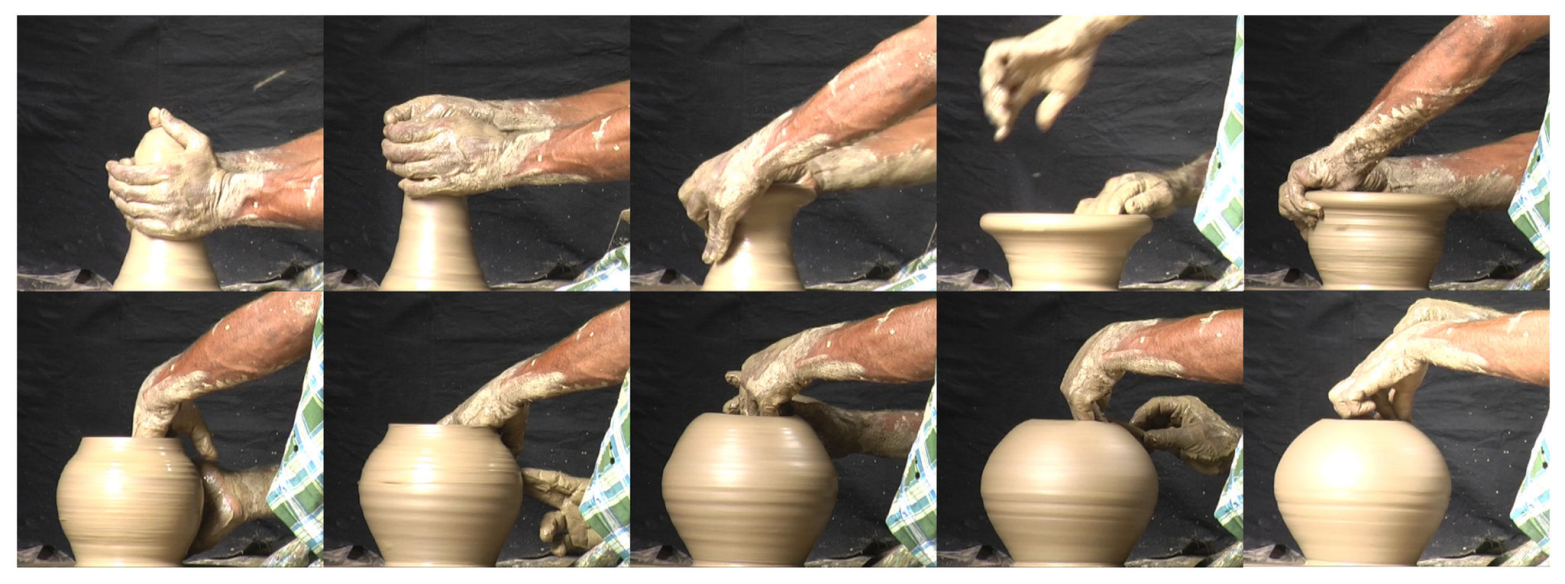
Examples of hand positions used during wheel-throwing. The images were extracted from a video recording of an Indian Multani potter throwing a 2.25 kg sphere. From left to right and top to bottom, the positions were coded as numbers 1, 27, 28, 4, 5, 7, 23, 17, 11, and 20, respectively (see Supporting Information S1).

In analysing motor skills it is useful to distinguish how the skill is performed (operation) from the result (outcome) of the skill. If cultural environments lead to specific cultural motor skills, do such operational differences give rise to differences in the outcomes? Here we address the relationships between the motor skill and its outcome by analysing pottery wheel-throwing in French and Indian cultural settings. To the best of our knowledge, this study is the first to experimentally examine both the operation and function of a given motor skill in different cultural settings. As a wide-spread, traditional and artisanal skill, wheel-throwing provides an excellent model for analysing the cultural transmission of motor skills. Starting with a formless lump of clay, the goal of wheel-throwing is to produce a vessel using a wheel rotating in the horizontal plane at speeds varying between 50 and 150 rotations/min [29,30]. Two main phases can be distinguished in the throwing process: during the pre-forming phase the potter centres the mass of clay on the wheel and subsequently sets the stage for the forming process by opening (hollowing) the centred lump of clay. During the forming phase thinning the clay walls brings out the initial form as the vessel rises from its base, while the finished object is acquired after a fast final shaping operation. In the throwing process potters successively deploy several distinctive hand positions for contact with the clay (see Figure 1) and a given hand position can be used at different moments [31]. Field observations by EG in France, Morocco, Azerbaijan, Turkey, India, and Palestinian territories suggested that these hand positions might be culturally dependent.

Our specific goal was to examine the ability of potters from distinct cultural settings (France and India) to reproduce a common model shape. We quantified the number and duration of the different hand positions utilised by the potters. In addition, we geometrically characterized the vessels produced in order to assess their degree of similarity. We predicted that the hand positions used by the potters (i.e. the operational aspect of motor skill) were influenced by the cultural background and would be different between the two groups. We therefore predicted that each group would manufacture pots (outcomes of the motor skills) characterized by culturally distinctive geometric features. Indeed, several cultural parameters could influence the ability of potters to reproduce a specific shape, such as the repetition of practice (i.e. training) for a specific repertoire of usual shapes and the omnipresence of cultural artefacts with specific shapes in the surrounding cultural environment. Assuming that the hand position repertories are indeed culturally specific, the possibility of convergent outcomes would open the way to a rich interpretative discussion.

## Materials and Methods

### Participants and cultural settings

Seven French and six Indian professional potters voluntarily participated in the experiment. The French potters lived and worked in different regions of France; whereas the Indian potters of the Multani community all lived and worked in the same Indian village (in the Uttar Pradesh region). All participants were right-handed and had a minimum of ten years of wheel-throwing experience (French: 30.3 ± 8.6 years; Multani: 16.5 ± 6.4 years). In the Indian setting, the pottery handicraft is still organized in a traditional way: the skill is learned within endogamous castes that produce standardized traditional objects in mass production [31–34]. In France, the social organization of pottery handicraft has evolved with the industrialization; the individual pottery apprenticeship has been transferred from family workshops to the public domain and the production design has become predominantly artistic. In addition to these distinctive social organizations, the instruments (wheels) and the raw materials (clays) also vary over the two cultural contexts. While the French potters used an electrical motor-driven wheel, the Indian potters used a kick wheel. The local clay used by the two groups differed in mechanical characteristics: indentation tests revealed a higher hardness in the French (2.6 kg/cm^2^) than in the Indian (1.7 kg/cm^2^) raw material.

The experiment was conducted in two pottery workshops, representative of the different cultural contexts. The French workshop was located in the Bourgogne area of France, while the Multani workshop was located in an Indian village of Uttar Pradesh (district of Bulandshar). Working in their natural conditions, potters were asked to reproduce a hollow sphere using two different quantities of clay (0.75 kg and 2.25 kg). This model shape was not a part of the everyday repertoire for either group. We chose the spherical shape because of its simple geometry (i.e. regularity of its curve) and because of its neutral (i.e. transcultural) value given its resemblance to several existing ceramic shapes in different places around the world. The model shape was presented as a 2D image without providing any indication of required absolute dimensions. The participants were simply instructed to faithfully reproduce the model shape and to throw vessels with the thinnest walls possible. The two experimental conditions (referred to as small and large spheres) were produced in five replicates; thus each participant produced a total of ten pots. Participants briefly practiced the task the day before the experiment.

### Data recording and analysis

The experimental sessions were videotaped and the image of each finished vessel was extracted from the films. Using Actogram® timing software we captured the sequence of hand positions used for throwing each pot, with measurements of the durations (seconds) spent in each position. A code was attributed to each specific hand position detected. In order to describe as objectively as possible the positions observed, we established a repertoire ethogram in which each position was exemplified by several pictures. A total of 62 different hand positions were identified (see Supporting Information S1). The non-shaping actions (e.g., to wet the clay) were also timed but were excluded from the total shaping duration. For each individual vessel thrown, we calculated the percentage of total shaping time (%Shap) for which each of the 62 hand positions was used. Then, for each potter and each of the two masses of clay used, we obtained an individual repertoire of %Shap defined as a vector of 62 values corresponding to the percentage time spent by this specific potter using each of the 62 hand positions. Given the fact that each potter used only part of the total hand position repertoire, several values in the vectors were equal to zero. 

From the images of the finished vessels, we extracted the 2D coordinates of the cross-sectional profiles by tracing them out on a Cintiq 21UX Wacom® tablet. The coordinates were converted from pixels to centimetres using a predetermined calibration factor. The coordinates were re-sampled to generate an equal number of points (256 in total) at regular height intervals along the y-axis and were finally smoothed with a low pass filter (see Supporting Information S2). As was to be expected [35], the vessels thrown with 2.25 kg of clay were larger than the vessels thrown with 0.75 kg of clay (see Table 1 for absolute dimensions).

**Table 1:** Absolute dimensions of the vessels thrown with the two clay masses by the French and Multani potters.

		French		Multani	
	Mass	0.75 kg	2.25 kg	0.75 kg	2.25 kg
H (cm)	Mean	12.0	18.5	11.0	17.0
	SD	1.2	2.0	1.3	1.5
B (cm)	Mean	9.6	14.3	8.9	13.2
	SD	1.0	1.4	0.6	1.1
A (cm)	Mean	8.4	11.5	7.2	9.5
	SD	0.9	1.0	0.8	0.7
MD (cm)	Mean	15.9	23.4	14.6	21.7
	SD	0.4	1.1	0.6	0.7
HMD (cm)	Mean	5.9	9.0	5.8	8.8
	SD	0.6	1.0	0.8	0.9

Group x Mass ANOVAs on the participant means revealed significant main effects of Mass on all absolute dimensions (all *F*s(1, 11) > 250, *p*s < 0.001). Main effects of Group were found for A (*F*(1, 11) = 14.5, *p* < 0.01) and MD (*F*(1, 11) = 19.2, *p* < 0.01). There were no significant Group x Mass interactions.

H: Height, B: Base, A: Aperture, MD: Maximal Diameter, HMD: Height of Maximal Diameter. Mean: Group mean, SD: Group standard deviation.

In order to capture the geometrical form, independent of the size, of the vessels produced, the analysis proceeded in several steps. First, the ensemble of profiles was submitted to an elliptical Fourier analysis (see [36] for further details). The Fourier coefficients were then normalized to the first harmonic to remove differences in size and orientation according to the method proposed by [37]. Finally, a Principal Component Analysis was performed on the normalized Fourier coefficients (including all groups and corresponding masses). As expected from the simple model shape, the first two PCs captured 96% of the variance (PC1 captured 94.4%, PC1 and PC2 cumulated captured 96.2%) (see Supporting Information S3 for details). The geometry of each vessel could therefore be adequately represented as a point in the two-dimensional PC1-PC2 space.

The effects of Mass (0.75 and 2.25 kg of clay) and Group (French and Multani) on the geometry of the thrown vessels were evaluated using a MANOVA (Multivariate Analysis of Variance) on PC1 and PC2. Operationally, this analysis tested whether Group and Mass influenced the mean location (i.e., the centroids of the distributions) of the vessels’ geometry in PC-space. Significant (α = 0.05) main effects and interactions were further explored using Newman-Keuls post-hoc tests.

### Ethics statement

The study consisted in non-invasive behavioural observations of potters in their habitual workshop. Potters gave informed written consent prior to participation and were paid for their participation according to the local rates of the profession. These observations were made in the framework of a Ph.D. project of Aix-Marseille University (France). According to the current French laws on the protection of persons in biomedical research (law No 88-1138, so-called Huriet-Serusclat law of the 20th December 1988, amended in 2004 - law of the 9th August 2004), such a protocol does not require the approval of an ethics committee. The study complies with the ethics guidelines provided by the National Consultative Ethics Committee of the French Centre National de la Recherche Scientifique (COMETS). The data were analysed anonymously. The cultural identification of the participants was simply their respective home countries.

## Results

### Hand positions

**Figure 2:**
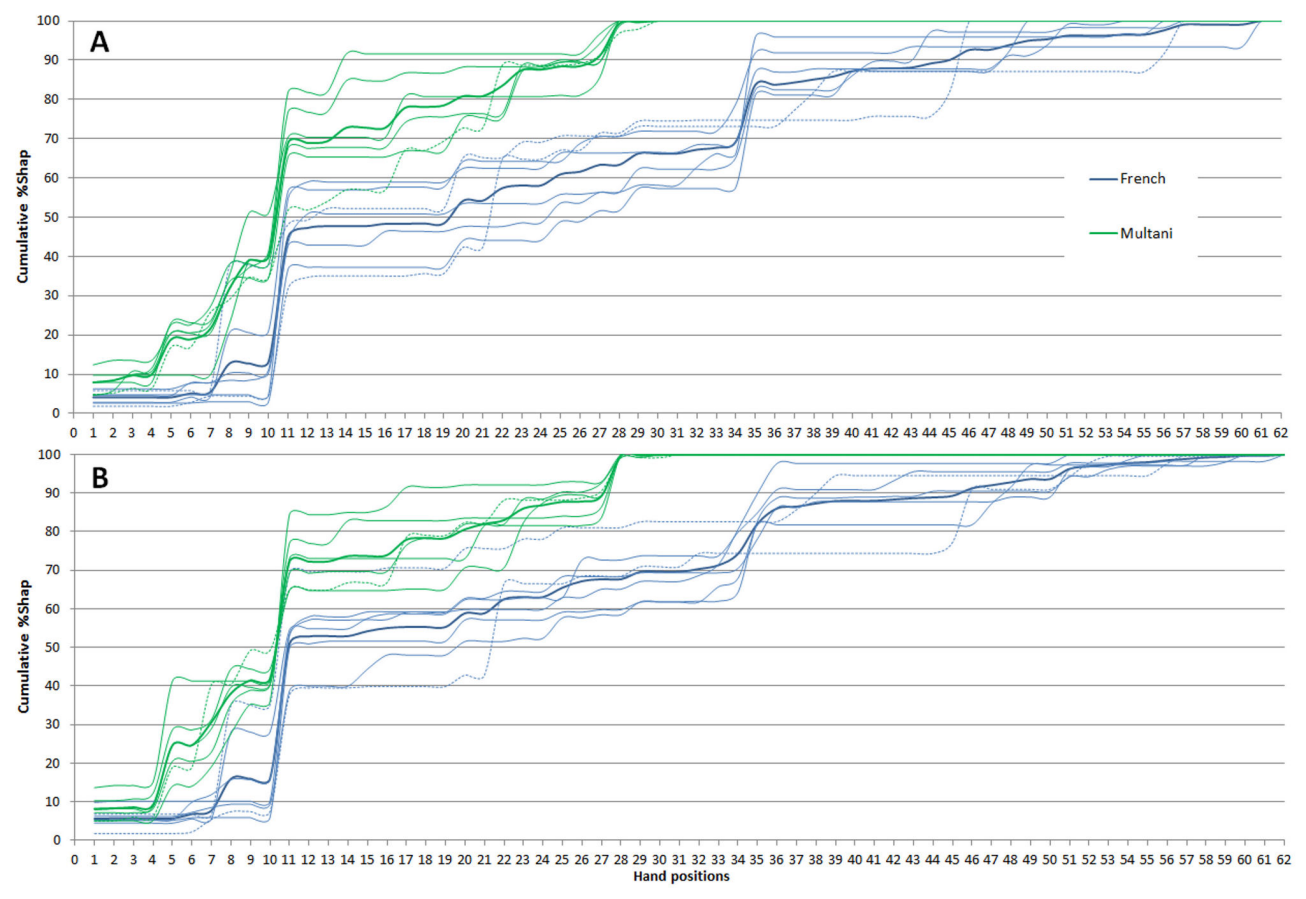
Cumulative percentage shaping time over the different hand positions. Individual (thin lines) and mean (bold lines) routes through the full hand position repertoire for the French (blue lines) and Indian Multani (green lines) potters. Panel A: Throwing small 0.75 kg spheres. Panel B: Throwing large 2.25 kg spheres. The dashed lines represent the potters who used individualistic repertories of %Shap hand positions.

The potter’s ‘route’ in producing the small and large spheres through the hand position repertoire is presented in Figure 2. This figure presents the cumulative percentage shaping time (%Shap) of hand positions used by each participant (thin lines) and the group average (bold lines). Inspection of this figure brings out several noteworthy observations.

Throwing with the two different masses revealed a considerable overlap in the hand positions deployed (Figures 2A and 2B). In fact, among the total of 62 different hand positions identified in this study, 46 (74%) were used for shaping the two masses. The repertoire for the small spheres included 50 hand positions, whereas the repertoire for the large spheres was slightly wider, including 58 hand positions. Only 4 hand positions were unique to the small spheres and 12 to the large spheres.

A striking result is the cultural group difference in the number of different hand positions used. Over the two masses, the French repertoire revealed 51 different hand positions while the Multani repertoire was limited to 28. Scrutiny of the routes followed by individual potters indicates that the French repertoire contained more idiosyncratic hand positions than the Multani repertoire. Of the 51 hand positions of the French repertoire, 31% (16/51 hand positions) were observed only in single potters, whereas 25% (7/28 hand positions) were idiosyncratic in the Multani repertoire. 

As can be observed from the bold lines (group averages) traced in Figure 2, certain hand positions were extensively used by one particular group. Particularly noticeable in this respect are positions 34, 35 and 36 for the French group and positions 5, 9, 14, and 28 for the Multani group. More precisely, among the 62 hand positions identified in the study, 34 were French-specific, whereas 11 were Multani-specific. Leaving aside the idiosyncratic hand positions, 20 non-idiosyncratic hand positions were identified as specific to the French potters and 7 to the Multani potters. On the other hand, the overlap between the repertories of the two groups involved 17 hand positions. Of these group-overlapping hand positions 12 were observed in at least two potters of each group, while the five others were idiosyncratic in one of the two groups (or in both groups).

In sum, analysis of the hand positions used by the two groups suggests a number of significant cultural differences. From the total of 62 different hand positions identified, 44% were culture-specific (20 French and 7 Multani) and only 27% were shared across cultures. Moreover, the extent of the hand position repertories varied over groups, with potters from the French group using more (82% of the total repertoire) different hand positions than potters from the Multani group (45% of the total repertoire). Finally, the French group also revealed relatively more (31%) idiosyncratic hand positions than the Multani group (25%).

**Figure 3:**
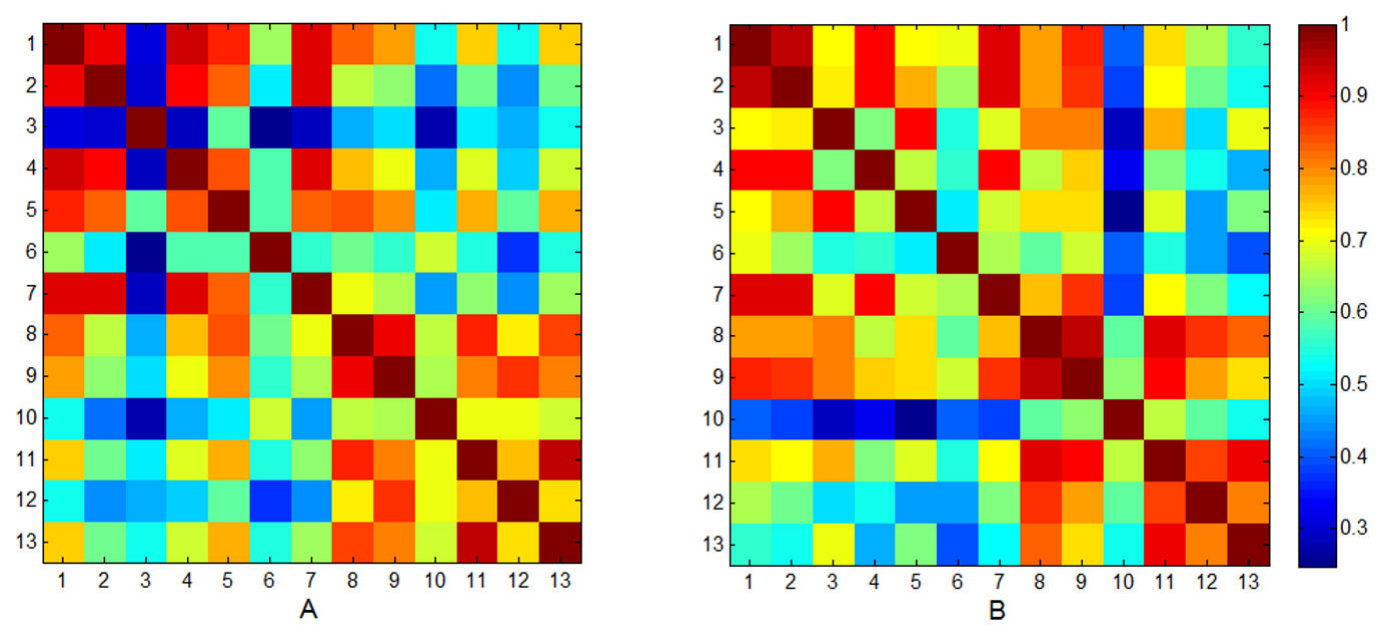
Correlation matrices for percentage shaping time over hand positions. Potters 1 to 7 constitute the French group and potters 8 to 13 constitute the Indian Multani group. The colour coding for correlation strength is presented on the right. Panel A: Throwing the small 0.75 kg spheres. Panel B: Throwing the large 2.25 kg spheres.

For all possible one-on-one combinations of the 13 potters, Figure 3 graphically presents the matrix of the Pearson correlation coefficients between the vectors of the individual repertories of %Shap hand positions, for each of the two masses separately. Note that quadrants 2 and 4 contain the matrices of the intra-group (French and Multani, respectively) correlation coefficients, while quadrants 1 and 3 contain the same but mirrored matrix of the inter-group correlation coefficients. These results demonstrate the combined influences of cultural origin and individual idiosyncrasies. Cultural influences were illustrated for both masses by stronger intra-group correlations compared to inter-group correlations: For the 0.75-kg mass the average (using Fisher-z transformation) intra-group correlations were 0.708 for the French group and 0.778 and for the Multani group, for an average inter-group correlation of 0.573 (*t*(61) = 2.39, *p* < 0.05 and *t*(55) = 5.12, *p* < 0.001, for the French and Multani groups respectively). For the 2.25-kg mass the average intra-group correlations were 0.780 for the French group and 0.808 for the Multani group, for an average inter-group correlation of 0.636 (*t*(61) = 3.44, *p* < 0.01 and *t*(55) = 3.96, *p* < 0.001, for the French and Multani groups respectively). These findings substantiate the cultural influence on hand position repertories: each group of potters demonstrated cultural homogeneity in the hand positions used and this was divergent from the other group. At the same time, the intra-group correlations were moderated by individual differences, with relatively more within-group variation in the French group. 

Visual inspection of Figure 3 revealed that two French potters (potters 3 and 6) diverged from their colleagues (especially for the 0.75-kg spheres), while one Multani potter (potter 10) diverged from his colleagues (for both masses). This results from the individualistic repertories of %Shap hand positions used by those three potters: French potters 3 and 6 did not use hand position 35 which was frequently used by all other French potters for both masses. In addition, for the 0.75-kg spheres, potter 3 frequently used positions 8, 20 and 13; he also used the idiosyncratic positions 37 and 39; potter 6 frequently used the idiosyncratic positions 22, 45 and 46 for both masses. In the Multani group, potter 10 used the idiosyncratic positions 22 and 26 for both masses and the idiosyncratic position 31 for 2.25 kg spheres (see Figure 2, dashed lines). 

### Geometry of the vessels thrown

**Figure 4:**
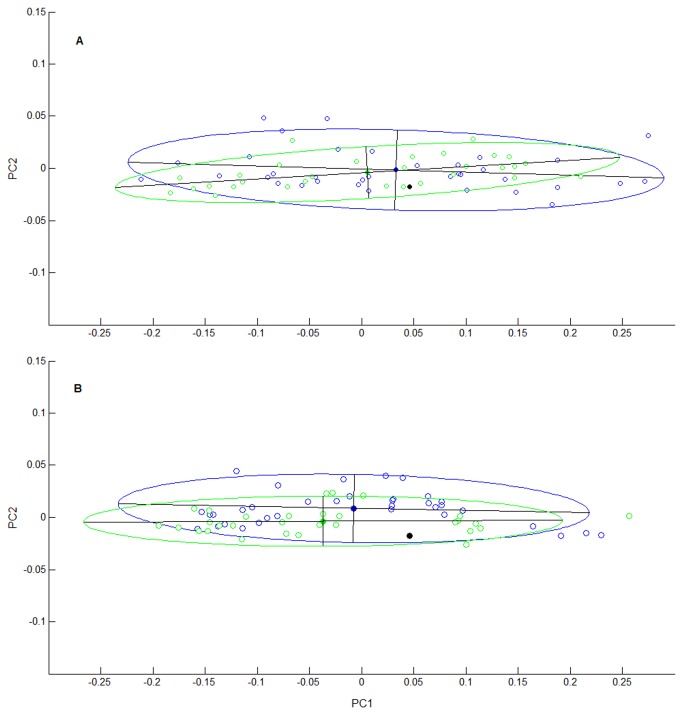
Geometrical distributions of vessels thrown. The geometrical space is formed by the first two principal components of the PCA performed on the coefficients of the elliptical Fourier analysis of the vessel outlines. Blue open markers represent all vessels thrown by the French potters; green open markers represent all vessels thrown by the Indian Multani potters. Using the same colour code, the 95%-confidence ellipses are superimposed, with their principal axes and centroids (solid markers). The solid black markers represent the model shape. Panel A: Small 0.75 kg spheres. Panel B: Large 2.25 kg spheres.

Figure 4 presents the geometrical characteristics of the vessels thrown by the French and Multani groups, for the small and large sphere conditions separately, in the two-dimensional PC-space. Visual inspection revealed that the distributions of small spheres (Figure 4A) were reasonably well centred on the geometry of the model shape. For the large spheres (Figure 4B), however, the centre of the distributions deviated to a certain extent from the model geometry. Surprisingly, for both masses, the French and Multani assemblages displayed a fair degree of similarity, in both the location and the orientation of the distributions in PC-space, indicating common shapes. The MANOVA confirmed the observations with respect to location in PC-space, as Mass was found exert a significant influence (*F*(2, 10) = 7.47, *p* < 0.01) while Group did not (*F*(2, 10) = 1.28, *ns*). Thus, the French and Indian potters produced vessels of equivalent shape when throwing the same amount of clay. However, for both groups, the shape of the vessels varied with the amount of clay used.

## Discussion

The cultural specificity of motor skills is part of the traditions of human societies [5-9]. In previous research the form and function of motor skills have not been addressed jointly [38,39]. Our experimental analysis of the potter’s skill of wheel-throwing bridges this gap.

Working under their habitual conditions, expert potters from distinct cultural settings (France and India) threw a series of vessels, with the goal of reproducing as closely as possible a 2D image of a sphere. As expected, results revealed a cultural influence on the operational aspects of the potters’ motor skill. 

Two points are noteworthy here. First, the two groups were distinguished by a significant number of culturally-specific hand positions, most likely moulded by the social environment in the course of learning. Second, it was apparent that the uniformity of the hand position repertoire was lower in the French group than in the Multani group. The French potters not only demonstrated a larger repertoire of hand positions, but also revealed more individualistic routes as well as a larger percentage of idiosyncratic hand positions. A noticeable example is the French potter 3 who, in throwing the small spheres, deployed a hand position repertoire that more strongly resembled that of the Indian rather than that of the French potters. This may be understood through the cultural differences in skill acquisition conditions: the French potters learned the skill in a more individualized, post-industrialized context of production whereas the Indian Multani potters were exposed to a traditional, family-centred context in which the reproduction of social models is more pervasive. 

The cultural differences in hand positions used did not give rise to noticeable differences in the shapes of the vessels produced. Both groups portrayed, for the two clay masses, shape equivalence notwithstanding small group differences in the size of the vessels thrown. Vessels thrown by the French potters were slightly bigger than those thrown by the Multani potters (Table 1). Importantly, the shape equivalence in both groups cannot be attributed to a statistical bias. Indeed, the geometries of the vessels of both groups did reveal systematic effects of the quantity of clay used which indicates that statistical sensitivity was in fact sufficient to reveal subtle but systematic differences. The thicker walls required by the softer clay used by the Indian potters most likely explain these size differences. As demonstrated in our earlier work [35], the transcultural effect of clay mass on the geometry of the vessels is due to mechanical factors. As can be seen in Table 1 (but see also Table 3 in [35]), larger masses of clay allow throwing bigger vessels. For both groups in the present study the vessels’ basic dimensions, such as height and maximal diameter, increased considerably with clay mass. However, if the bigger vessels had the exact same geometrical shape (i.e. homothetic scaling), they would inevitably undergo stronger internal mechanical stresses and hence run a larger risk of collapse [35]. Mechanical optimization thus requires adaptations in shape, which for a sphere can be obtained, for example, by reducing the vessel’s maximal diameter relative to its height. Given the fact that mechanical constraints are transcultural, it is not surprising that all expert potters, whether they were French or Indian, modulated the shape of their pots in relation to their size. A final remark concerns the differences observed in the clay used: French potters threw somewhat harder clay using an electrically-driven wheel while Multani potters threw somewhat softer clay using a foot-driven kick wheel. On the basis of the present data we cannot evaluate the effects of these culture-specific material differences. 

To conclude, French and Indian potters demonstrated clear differences in the operational aspects of their wheel-throwing skill, as revealed by the analysis of the hand positions deployed when reproducing a spherical model shape. In spite of such operational differences, the two cultural groups not only produced vessels of equivalent shape but also demonstrated comparable mechanical optimization when using larger clay masses. 

These findings, especially that of potential shape equivalence, have implications for the domain of archaeology. The shapes of artefacts, in particular ceramics, are classically used to produce typological classifications as a means for differentiating between cultures. Similarity of shape is interpreted as an indicator of cultural and regional uniformity [40-43]. The current study introduces a cautionary caveat to that assumption. Here we show that wheel-thrown ceramic vessels characterized by an equivalent shape could have been produced by potters belonging to distinct cultural settings with culturally distinct motor skills. Of course, one cannot (and certainly should not) infer that, for a given skill, all culturally-distinct motor skills systematically lead to equivalent outcomes. It is reasonable to assume that the culturally-specific hand positions used by both groups of expert potters were tailored to the production of their respective daily repertoire. Our results indicate that both cultural sets of hand positions are suitable for the production of vessels with a simple and culturally-neutral spherical shape. We emphasize that these findings do not imply that similar results should be expected either for vessels produced using other pottery techniques (e.g., coiling) or for wheel-thrown vessels with more complex, challenging and/or culturally-specific shapes. All that we may conclude from the present study is that motor equivalence is in principle possible. Consequently, to provide direct evidence for the origins of wheel-thrown ceramic vessels, the analysis of their shapes should be complemented with other techniques such as petrographic, chemical, residual, and potmark analyses [44-47]. 

## Conclusion

The differences in hand positions used by expert potters from two different cultural settings indicate cultural influence on motor skills. However, operationally different motor skills did not give rise to differences in the shapes of the vessels. In reproducing a spherical model, the vessels thrown by both groups of potters were geometrically equivalent. Independent of cultural setting, potters compensated for an increase in the mass of clay by modifying the vessels’ shape, presumably for mechanical optimisation reasons. As a wide-spread, traditional and artisanal skill, wheel-throwing provides an excellent model for analysing the cultural transmission of motor skills. More experiments are required to analyse the interrelationship between the cultural hand positions and the final geometry of different ceramic shapes.

## Supporting Information

Supporting Information S1 **Hand positions repertoire.** Photographs of the 62 hand positions identified in the French and Indian Multani groups.
(PDF)

Supporting Information S2 **Model shape and 2D cross-sectional profiles of the vessels thrown by individual potters.** Blue lines represent the 0.75-kg vessels; red lines represent the 2.25-kg vessels. Potters 1 to 7 constitute the French group and potters 8 to 13 constitute the Indian Multani group.
(PDF)

Supporting Information S3 **Details of the Principal Components Analysis of the Elliptical Fourier Coefficients.** The first sheet of the document contains the 15 pairs of Elliptical Fourier Coefficients for all the vessel profiles (groups and masses combined); the second sheet contains the percentages of variance explained by the first 10 PCs, the cumulative percentages of variance explained by the first 10 PCs, the Eigenvalues for the first 4 PCs, and the Eigenvectors (factor loadings) for the 15 pairs of Elliptical Fourier Coefficients.
(XLS)
