# Supplementary material for: How Can Ten Fingers Shape a Pot? Evidence for Equivalent Function in Culturally Distinct Motor Skills
Source: PLoS One. 2013 Nov 27;8(11):e81614. doi: 10.1371/journal.pone.0081614 (PMC3842241; doi:10.1371/journal.pone.0081614)

Hand Position Repertoire

In this study we analyzed the hand positions of the potter all along the shaping process. A hand position is the way the potter places his fingers on the lump of clay for each gesture. These hand positions are the only observable element of skill which could guide the novice during the apprenticeship. In fact, expert potters often encourage the novice to observe and copy their hand positions. Four main operations can be distinguished in the shaping process: the centering, the hollowing, the thinning and the final shaping. Generally, a specific hand position is used for a particular operation. The same position can be used by several potters and also for the same operation.

We have systematically observed (on video) the hand positions used by 13 expert potters (seven French and six Indian Multani) producing 10 sphere shaped vessels (five small and five large) in their traditional way (i.e. using their habitual wheel and their regular clay raw material). We have assigned a code to each position identified (N=62 distinct hand positions). The following table summarizes for each hand position: vessel size (small vs. large pots), artisans (French vs. Multani), and idiosyncratic position or no. The red color indicates the idiosyncratic hand positions (i.e. hand position used by a single potter in one of the two groups).

hand position	small sphere	large sphere	French	Multani	hand position	small sphere	large sphere	French	Multani
1	x	x	x	x	32	x	x	x	
2	x	x	x	x	33	x	x	x	
3	x	x		x	34	x	x	x	
4	x	x		x	35	x	x	x	
5	x	x		x	36		x	x	
6	x	x	x		37	x	x	x	
7	x	x	x	x	38	x	x	x	
8	x	x	x	x	39	x	x	x	
9	x	x		x	40	x	x	x	
10	x	x	x	x	41	x		x	
11	x	x	x	x	42	x	x	x	
12	x	x	x		43	x	x	x	
13	x	x	x	x	44	x	x	x	
14	x	x		x	45	x	x	x	
15		x	x		46	x	x	x	
16	x	x	x	x	47		x	x	
17	x	x	x	x	48	x	x	x	
18	x	x	x	x	49	x	x	x	
19	x			x	50	x		x	
20	x	x	x	x	51	x	x	x	
21		x		x	52		x	x	
22	x	x	x	x	53		x	x	
23	x	x	x	x	54	x	x	x	
24	x	x		x	55		x	x	
25	x	x	x	x	56	x	x	x	
26	x	x	x	x	57	x	x	x	
27	x	x	x	x	58		x	x	
28	x	x		x	59		x	x	
29	x	x	x	x	60		x	x	
30	x	x		x	61	x		x	
31		x		x	62		x	x	
					**total**	**50**	**58**	**51**	**28**
Idiosyncratic Positions					**total**			**16**	**7**

Hand position 1

hand position	small sphere	large sphere	French	Multani
1	x	x	x	x

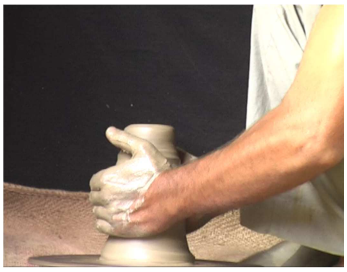
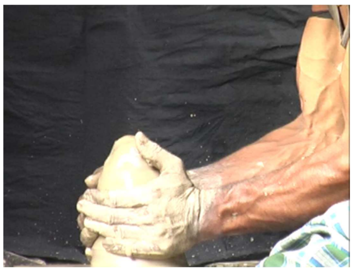
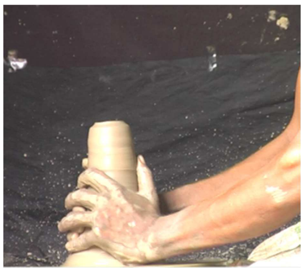
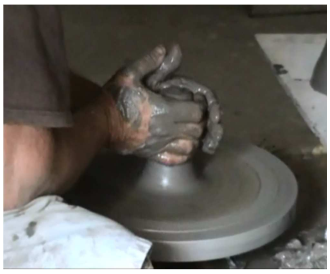
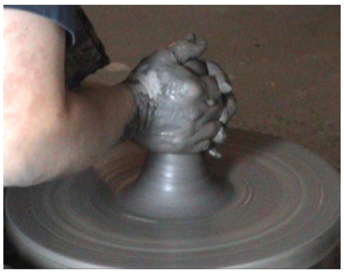

Hand position 2

hand position	small sphere	large sphere	French	Multani
2	x	x	x	x

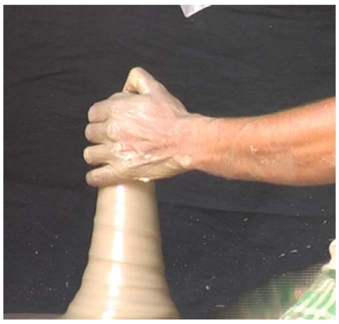
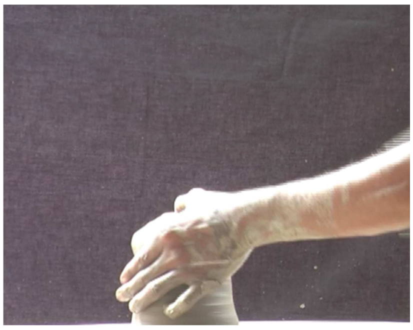
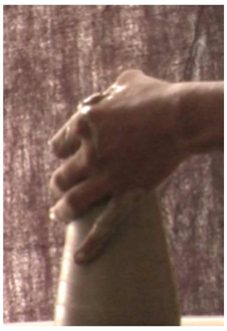
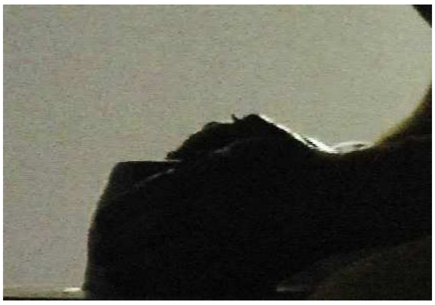

Hand position 3

hand position	small sphere	large sphere	French	Multani
3	x	x		x

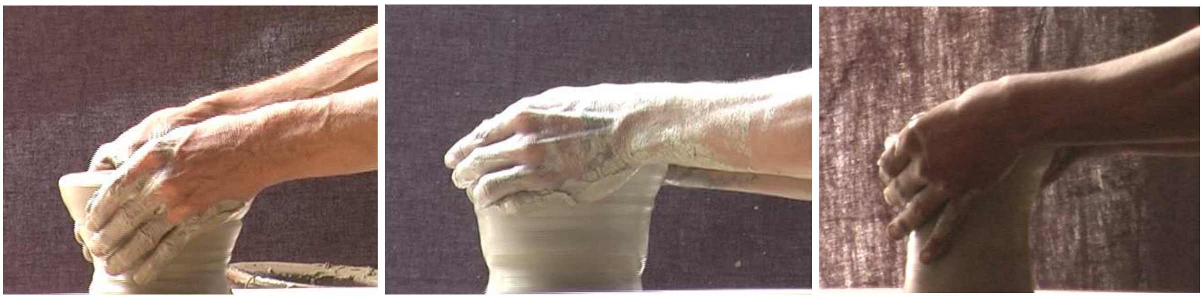

Hand position 4

hand position	small sphere	large sphere	French	Multani
4	x	x		x

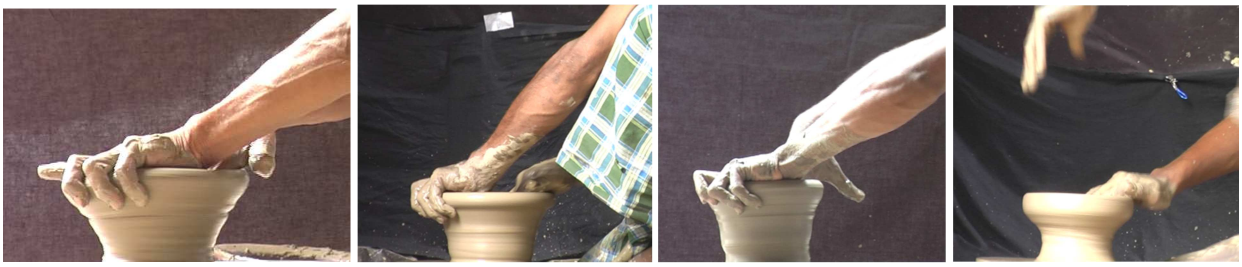

Hand position 5

hand position	small sphere	large sphere	French	Multani
5	x	x		x

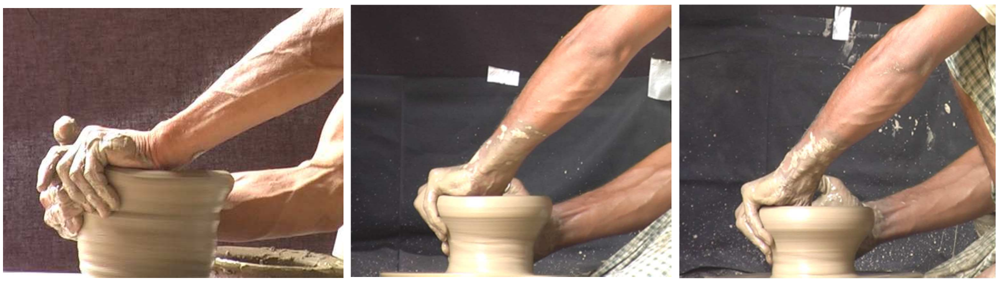

Hand position 6

hand position	small sphere	large sphere	French	Multani
6	x	x	x	

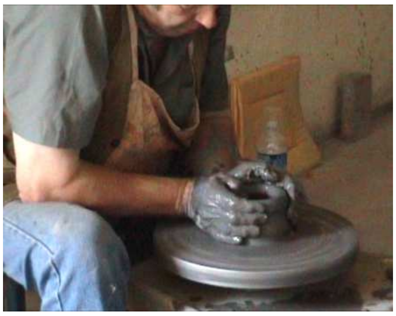
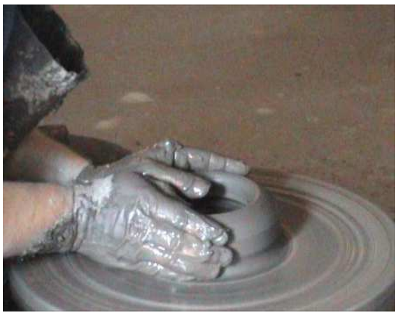
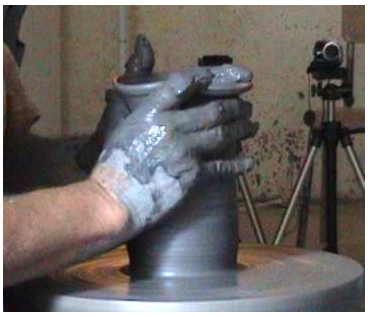
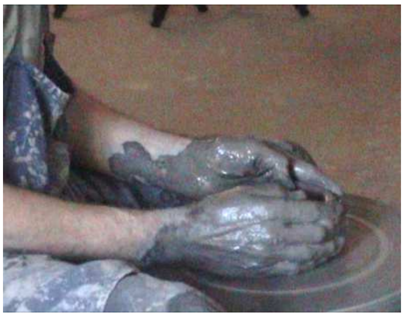

Hand position 7

hand position	small sphere	large sphere	French	Multani
7	x	x	x	x

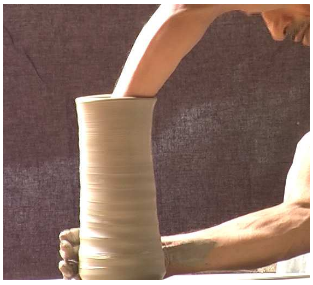
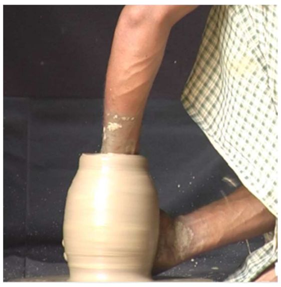
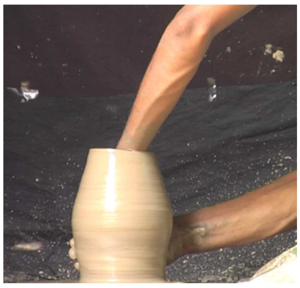
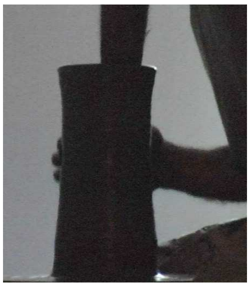
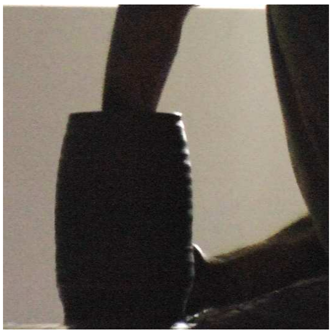

Hand position 8

hand position	small sphere	large sphere	French	Multani
8	x	x	x	x

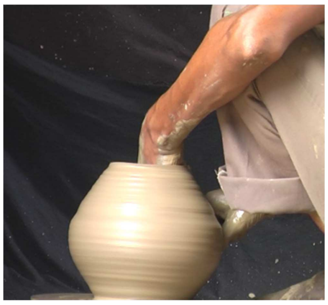
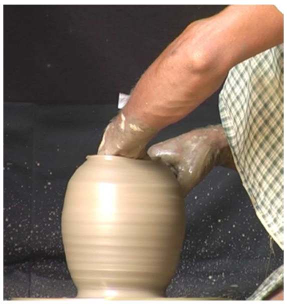
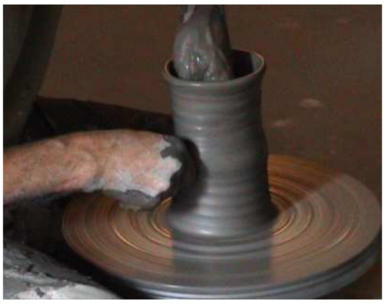

Hand position 9

hand position	small sphere	large sphere	French	Multani
9	x	x		x

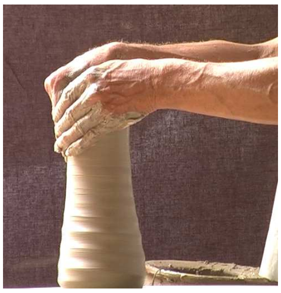
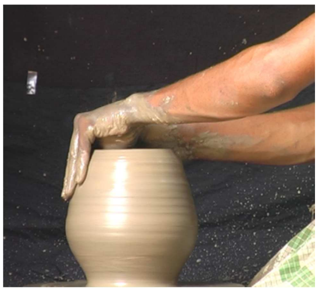
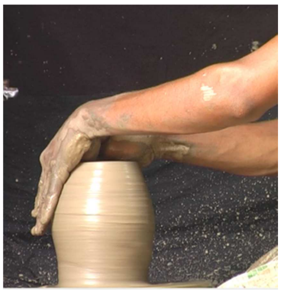

Hand position 10

hand position	small sphere	large sphere	French	Multani
10	x	x	x	x

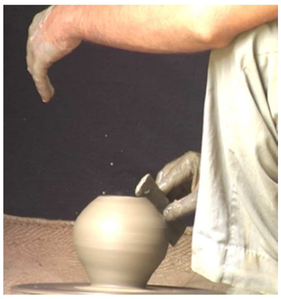
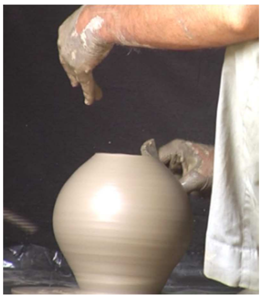
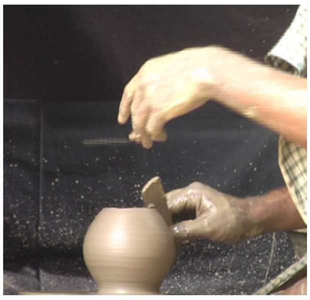
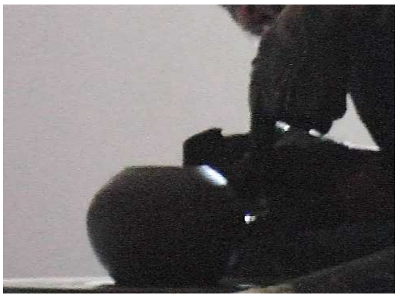

Hand position 11

hand position	small sphere	large sphere	French	Multani
11	x	x	x	x

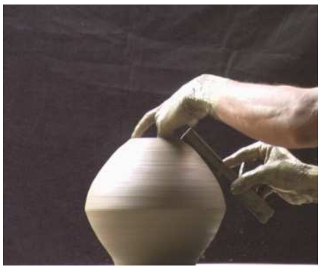
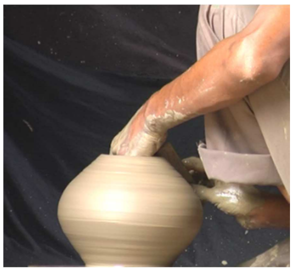
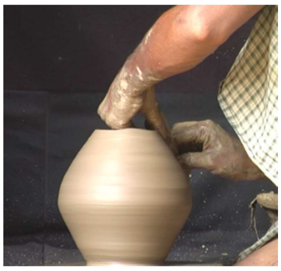
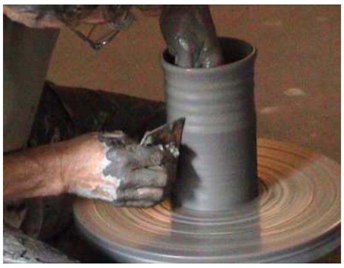
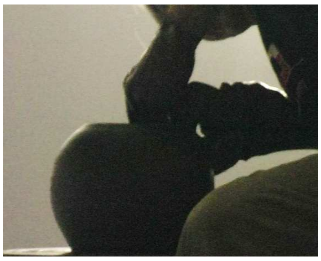

Hand position 12

hand position	small sphere	large sphere	French	Multani
12	x	x	x	

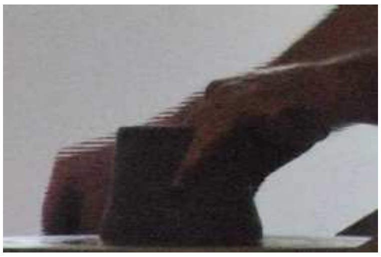
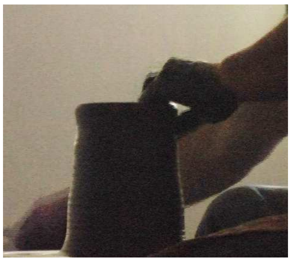

Hand position 13

hand position	small sphere	large sphere	French	Multani
13	x	x	x	x

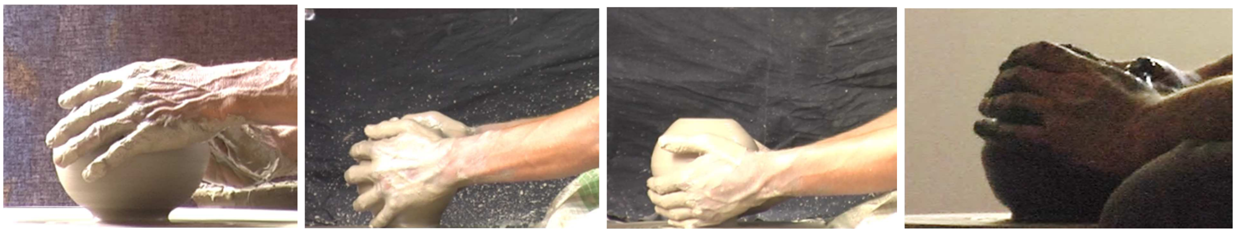

Hand position 14

hand position	small sphere	large sphere	French	Multani
14	x	x		x

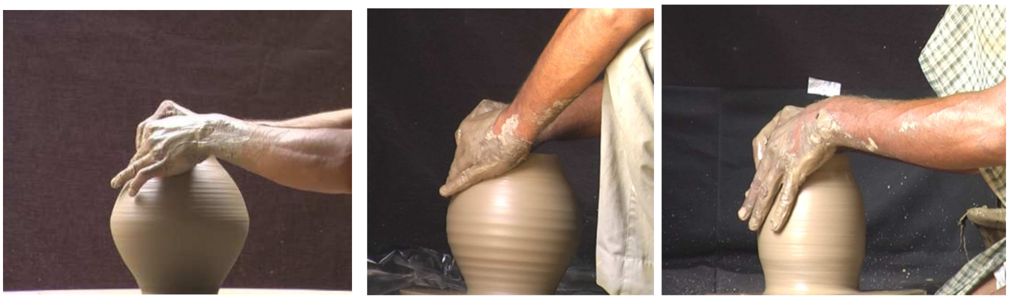

Hand position 15

hand position	small sphere	large sphere	French	Multani
15		x	x	

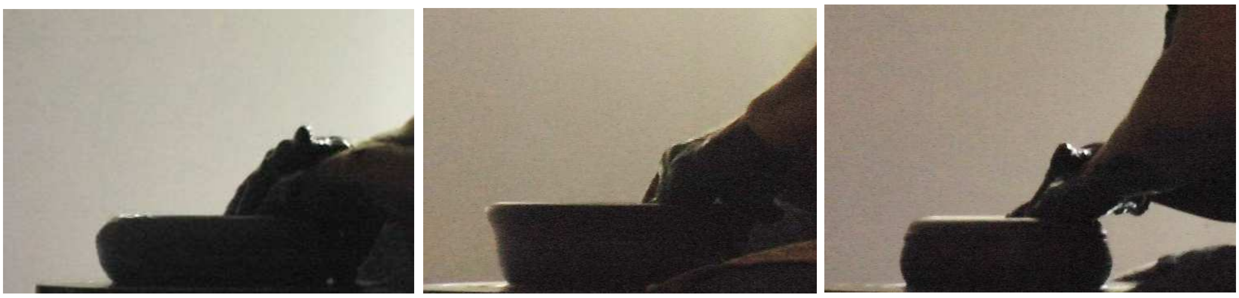

Hand position 16

hand position	small sphere	large sphere	French	Multani
16	x	x	x	x

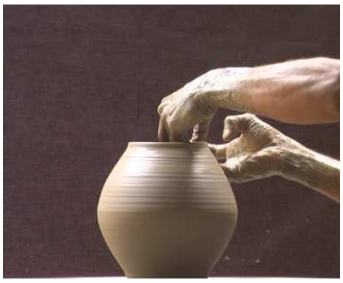
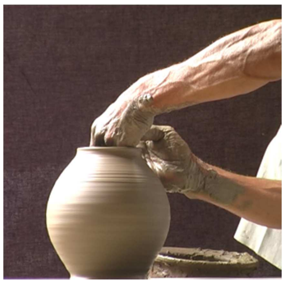
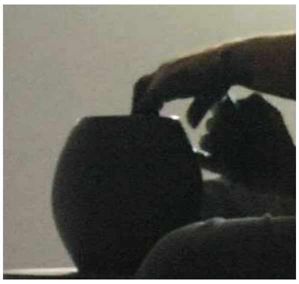

Hand position 17

hand position	small sphere	large sphere	French	Multani
17	x	x	x	x

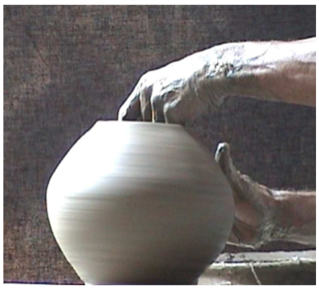
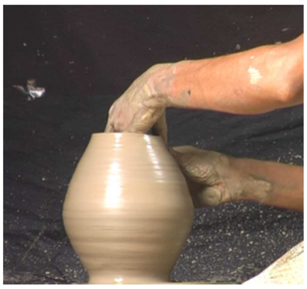
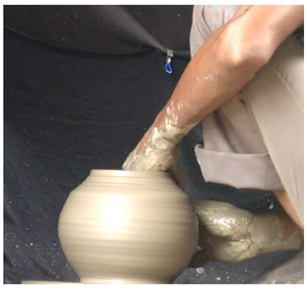
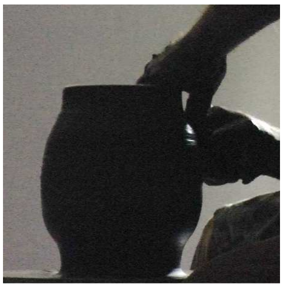

Hand position 18

hand position	small sphere	large sphere	French	Multani
18	x	x	x	x

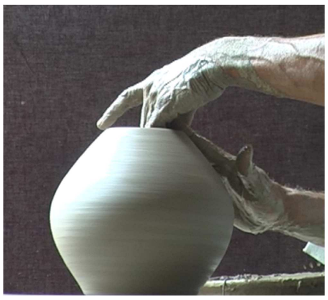
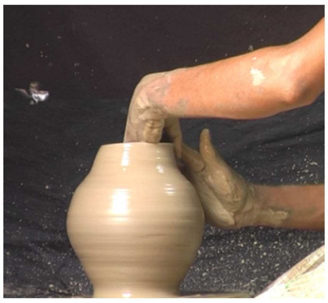
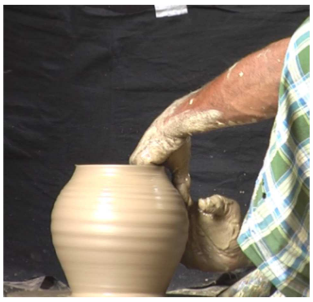
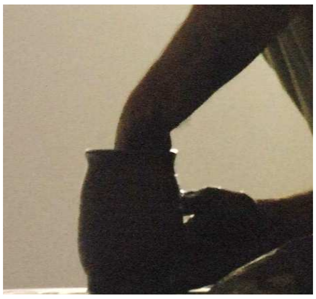

Hand position 19

hand position	small sphere	large sphere	French	Multani
19	x			x

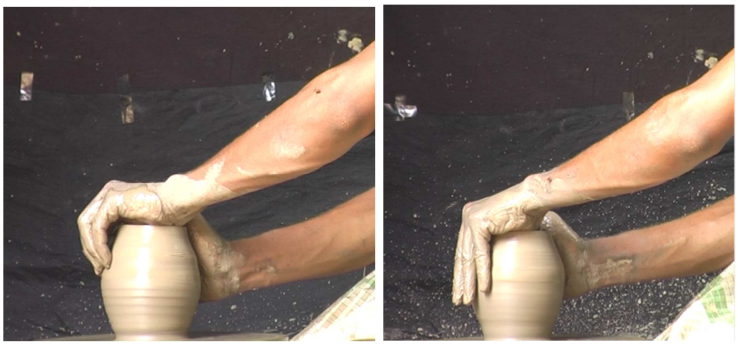

Hand position 20

hand position	small sphere	large sphere	French	Multani
20	x	x	x	x

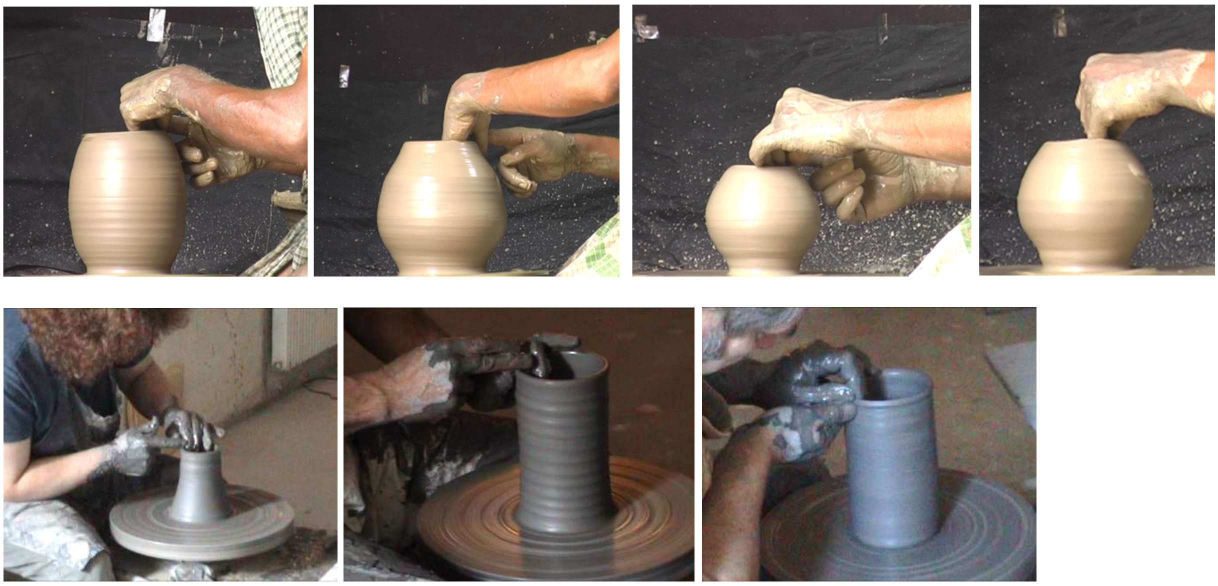

Hand position 21

hand position	small sphere	large sphere	French	Multani
21		x		x

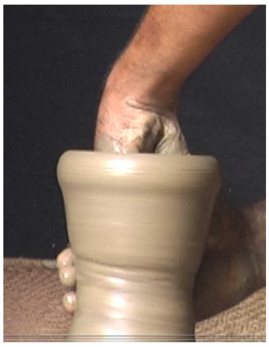
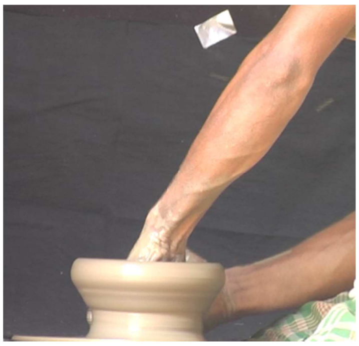

Hand position 22

hand position	small sphere	large sphere	French	Multani
22	x	x	x	x

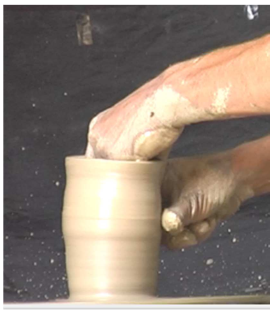
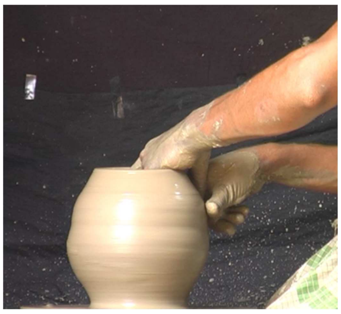
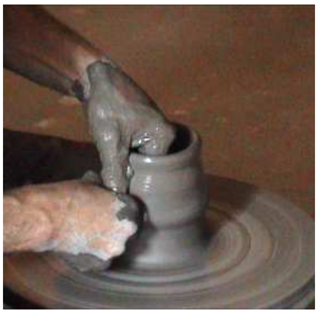

Hand position 23

hand position	small sphere	large sphere	French	Multani
23	x	x	x	x

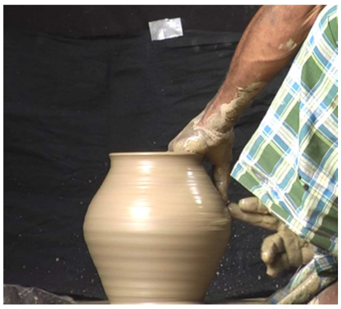
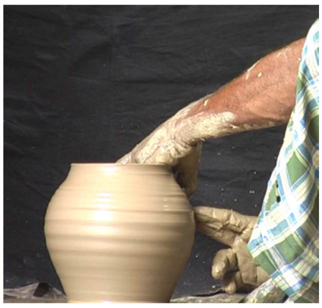
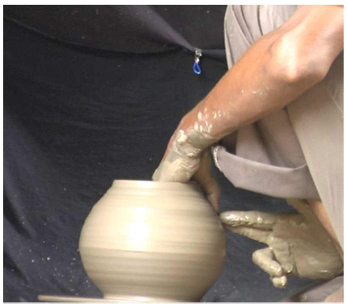

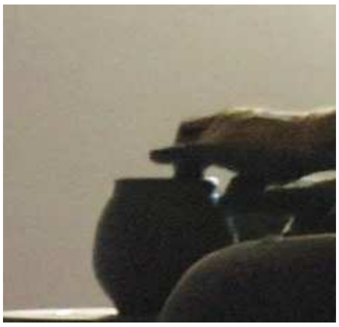
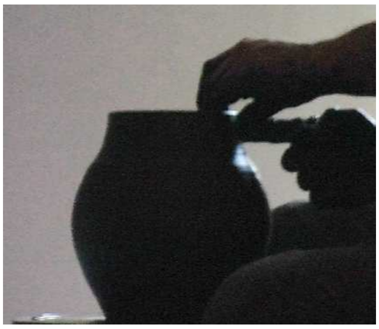

Hand position 24

hand position	small sphere	large sphere	French	Multani
24	x	x		x

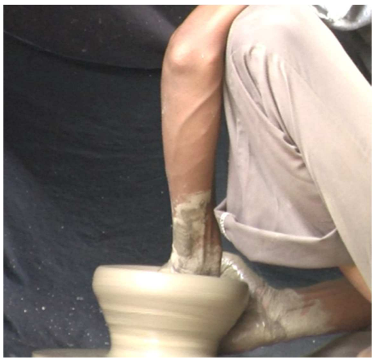
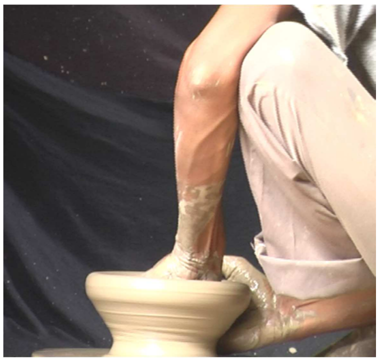

Hand position 25

hand position	small sphere	large sphere	French	Multani
25	x	x	x	x

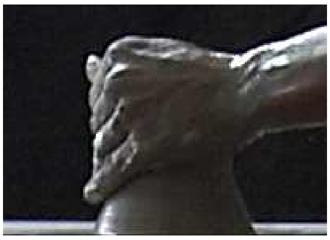
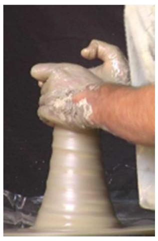
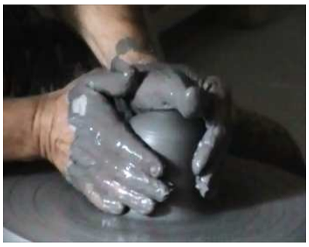
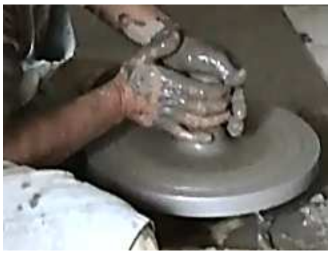

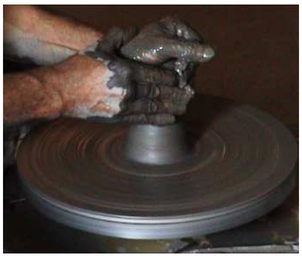

Hand position 26

hand position	small sphere	large sphere	French	Multani
26	x	x	x	x

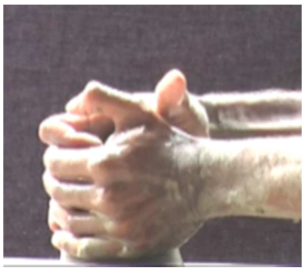
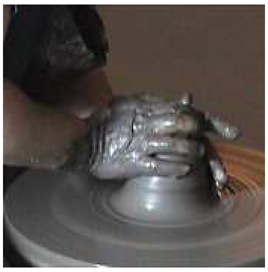
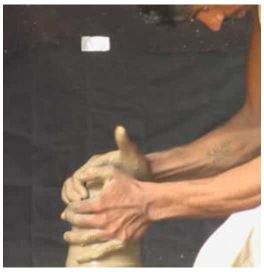

Hand position 27

hand position	small sphere	large sphere	French	Multani
27	x	x	x	x

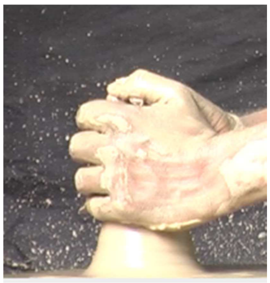
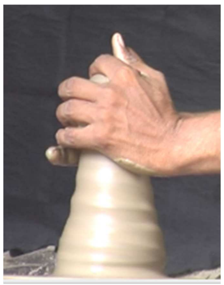
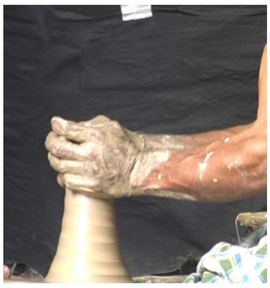
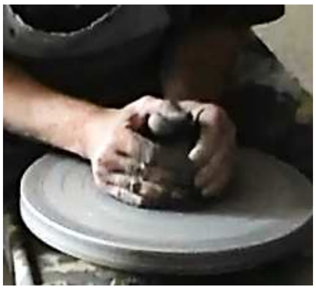

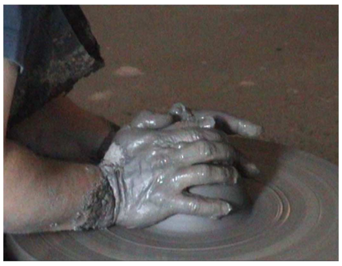

Hand position 28

hand position	small sphere	large sphere	French	Multani
28	x	x		x

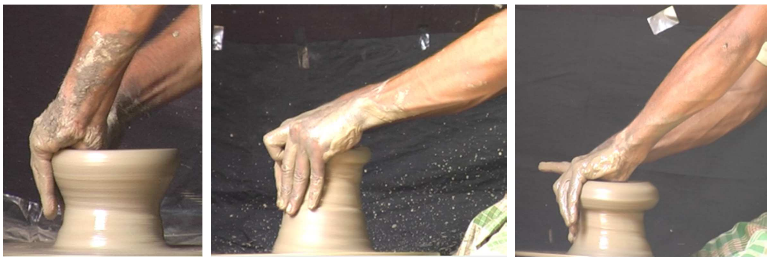

Hand position 29

hand position	small sphere	large sphere	French	Multani
29	x	x	x	x

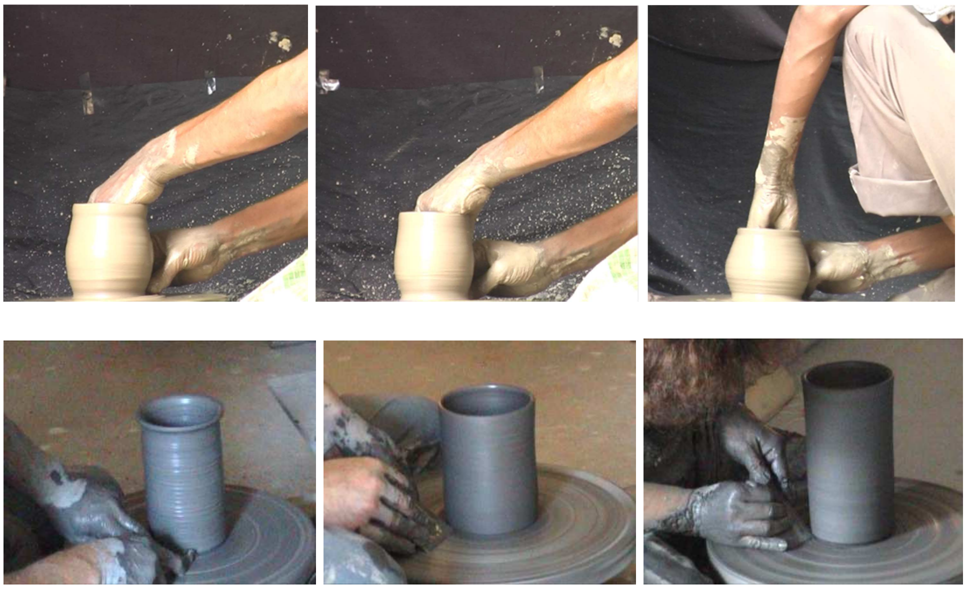

Hand position 30

hand position	small sphere	large sphere	French	Multani
30	x	x		x

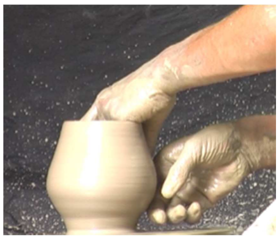
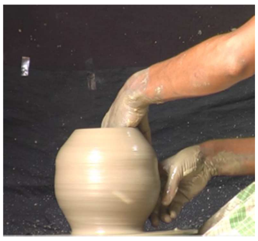

Hand position 31

hand position	small sphere	large sphere	French	Multani
31		x		x

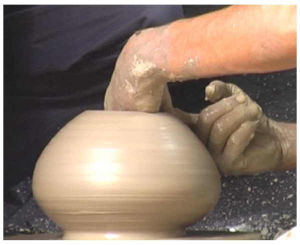

Hand position 32

hand position	small sphere	large sphere	French	Multani
32	x	x	x	

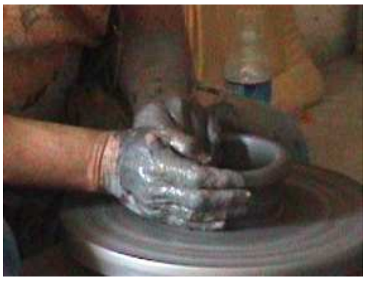
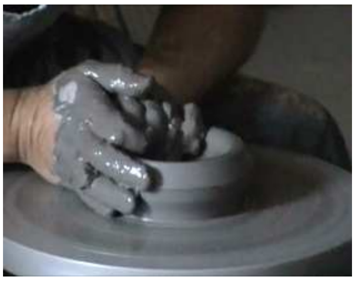
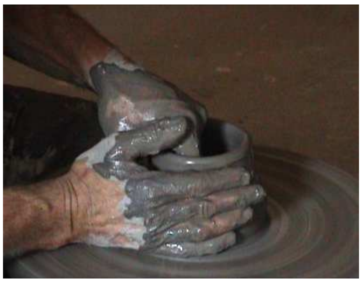

Hand position 33

hand position	small sphere	large sphere	French	Multani
33	x	x	x	

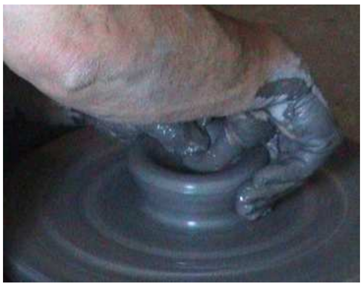
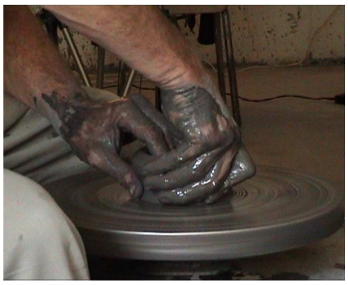
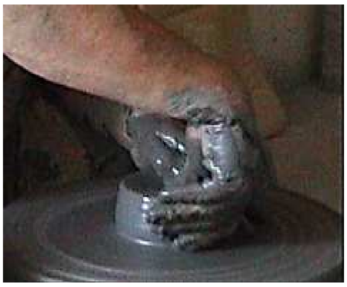

Hand position 34

hand position	small sphere	large sphere	French	Multani
34	x	x	x	

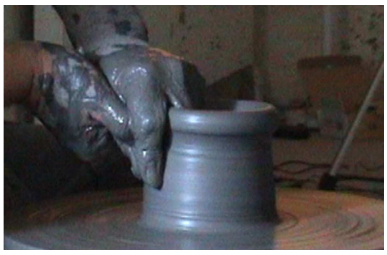
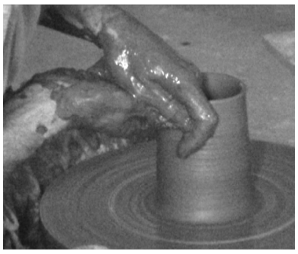
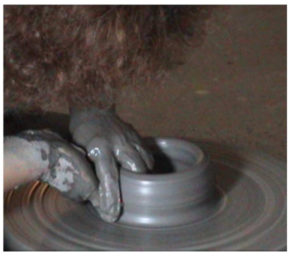
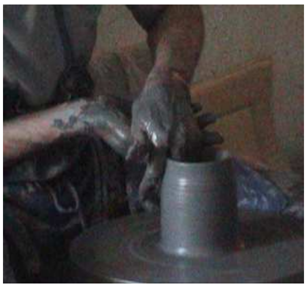
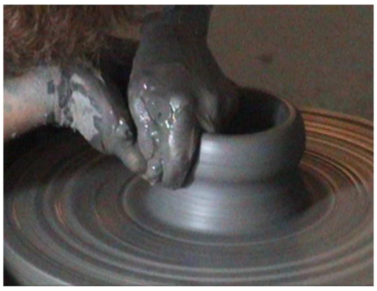

Hand position 35

hand position	small sphere	large sphere	French	Multani
35	x	x	x	

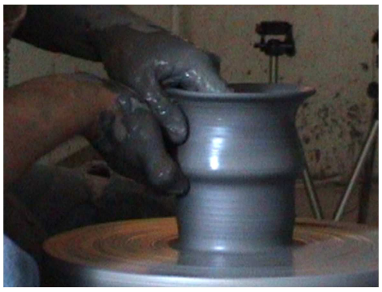
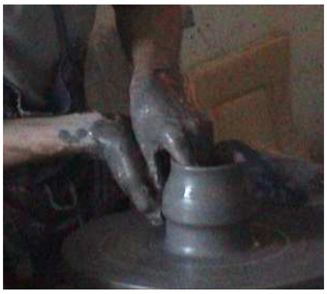
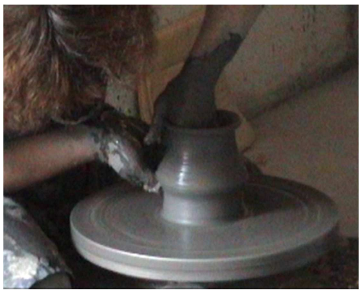
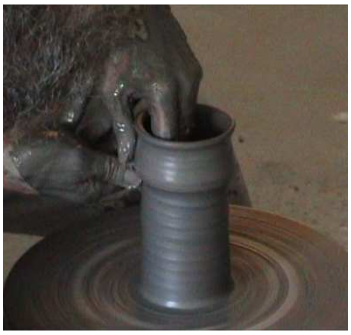
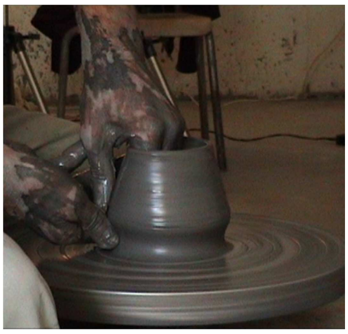

Hand position 36

hand position	small sphere	large sphere	French	Multani
36		x	x	

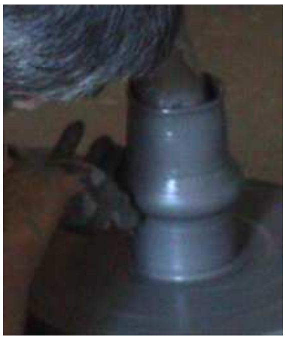
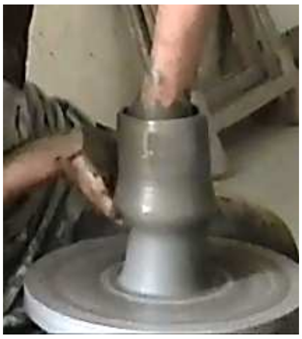
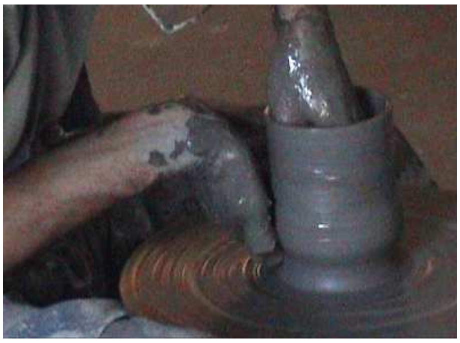
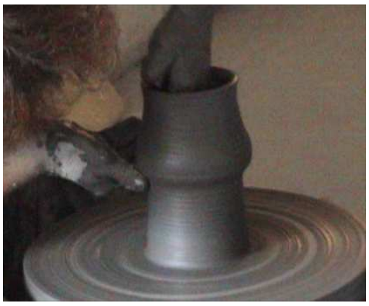

Hand position 37

hand position	small sphere	large sphere	French	Multani
37	x	x	x	

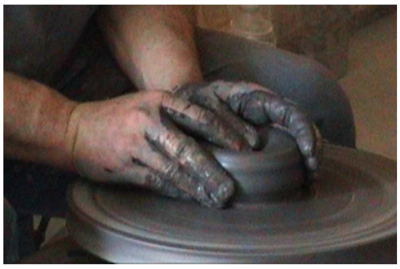
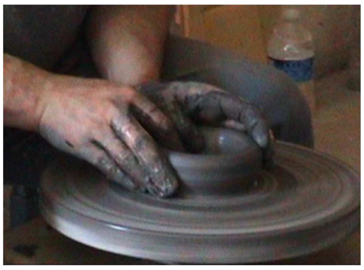

Hand position 38

hand position	small sphere	large sphere	French	Multani
38	x	x	x	

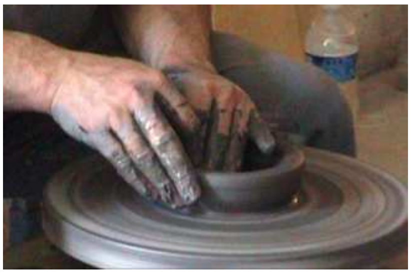
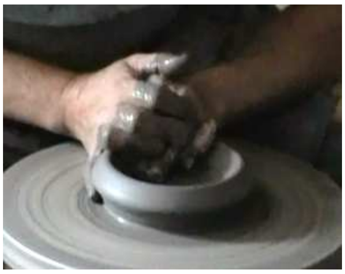
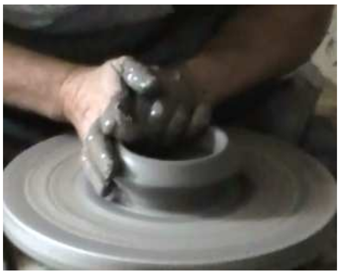

Hand position 39

hand position	small sphere	large sphere	French	Multani
39	x	x	x	

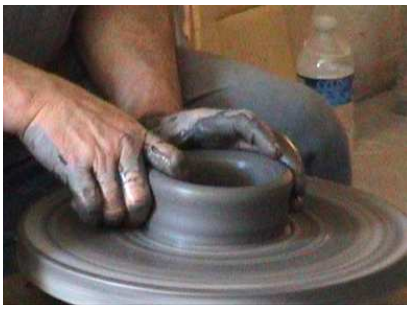
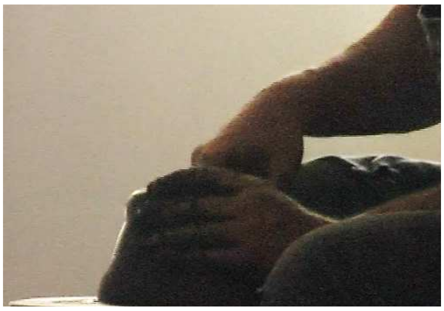

Hand position 40

hand position	small sphere	large sphere	French	Multani
40	x	x	x	

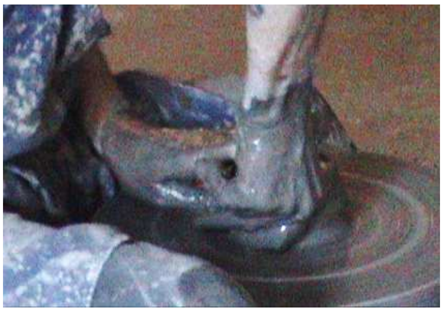
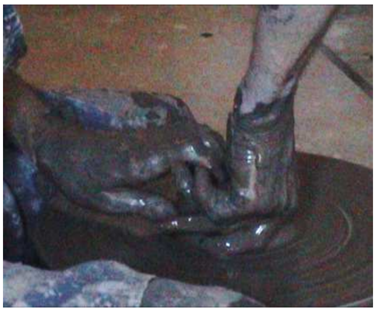

Hand position 41

hand position	small sphere	large sphere	French	Multani
41	x		x	

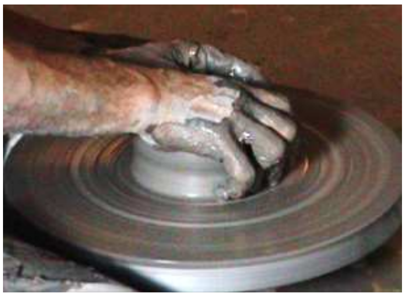
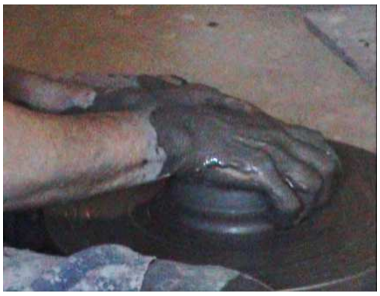

Hand position 42

hand position	small sphere	large sphere	French	Multani
42	x	x	x	

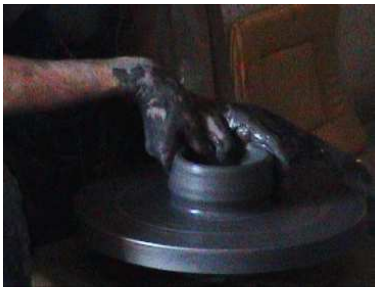
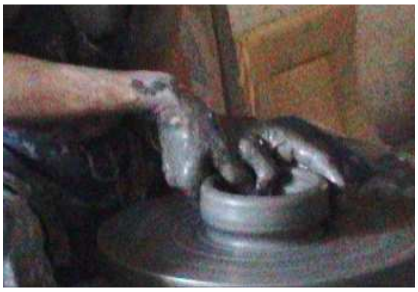

Hand position 43

hand position	small sphere	large sphere	French	Multani
43	x	x	x	

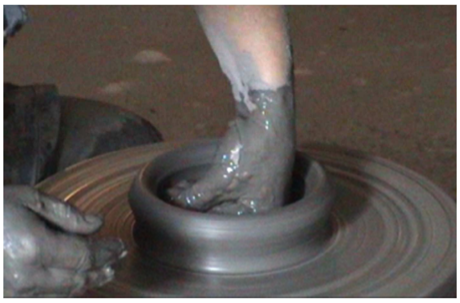
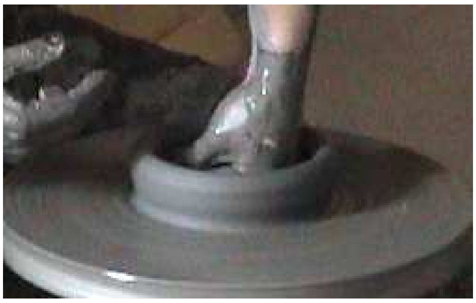

Hand position 44

hand position	small sphere	large sphere	French	Multani
44	x	x	x	

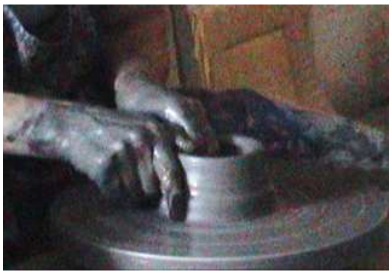
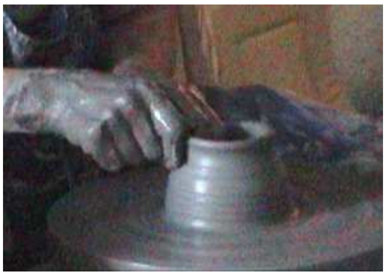
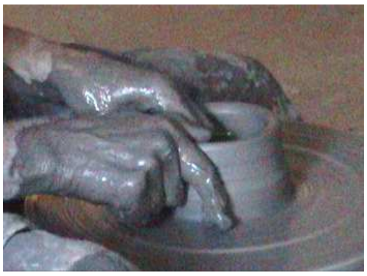

Hand position 45

hand position	small sphere	large sphere	French	Multani
45	x	x	x	

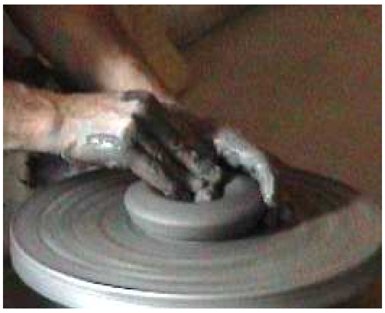
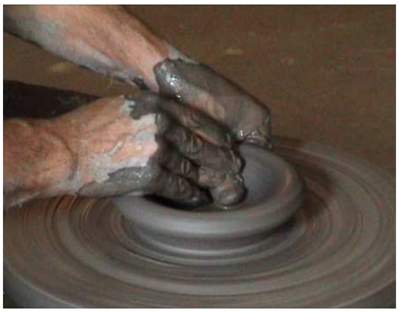

Hand position 46

hand position	small sphere	large sphere	French	Multani
46	x	x	x	

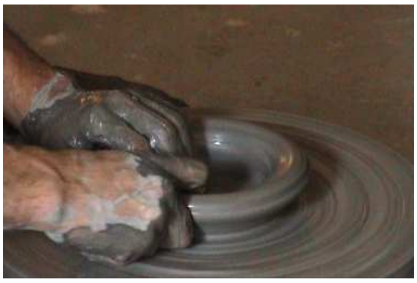
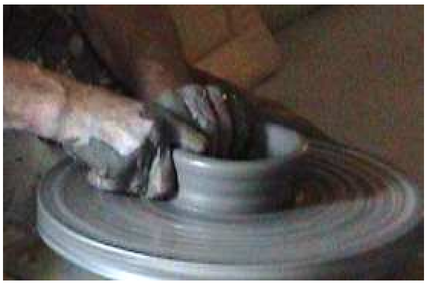
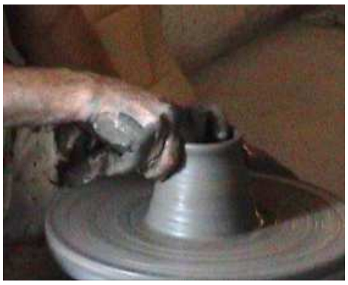

Hand position 47

hand position	small sphere	large sphere	French	Multani
47		x	x	

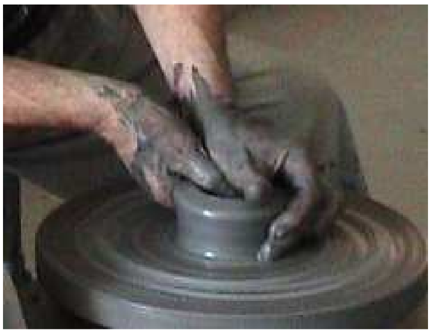
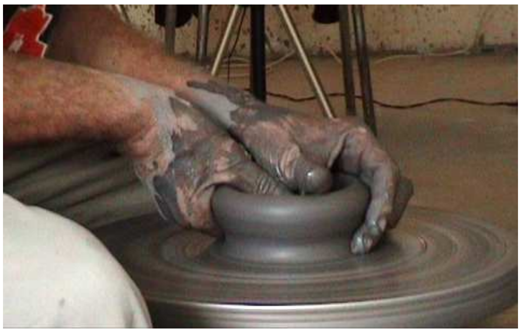

Hand position 48

hand position	small sphere	large sphere	French	Multani
48	x	x	x	

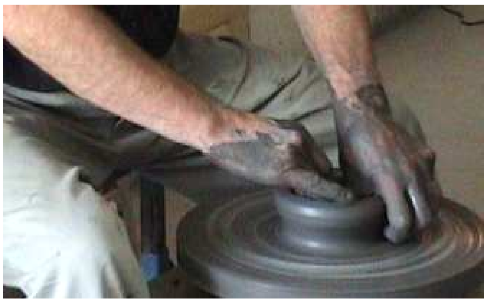
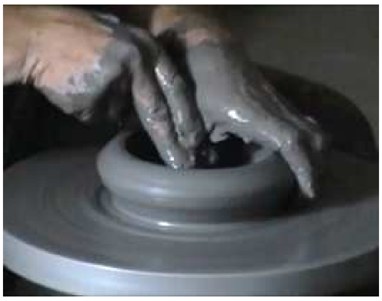
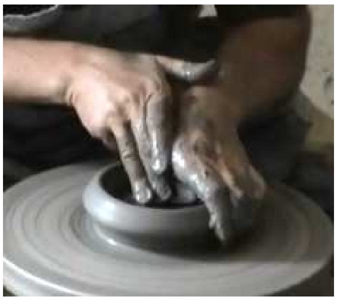

Hand position 49

hand position	small sphere	large sphere	French	Multani
49	x	x	x	

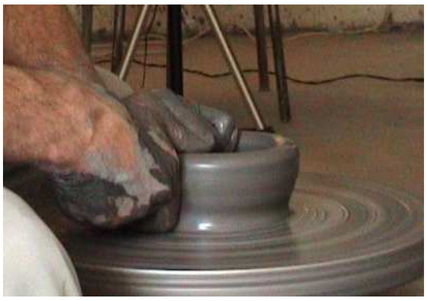

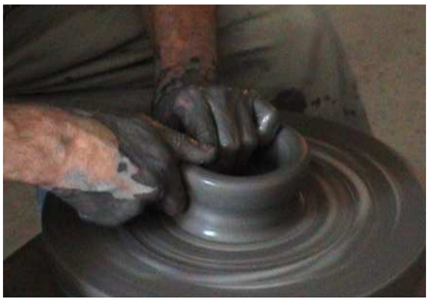

Hand position 50

hand position	small sphere	large sphere	French	Multani
50	x		x	

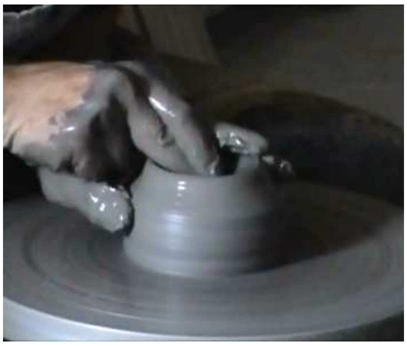

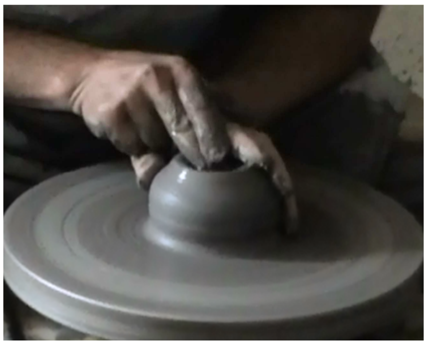

Hand position 51

hand position	small sphere	large sphere	French	Multani
51	x	x	x	

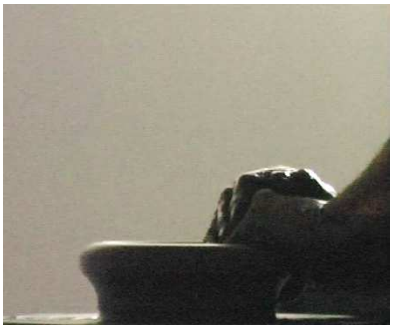

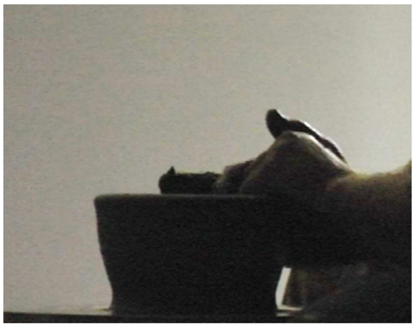

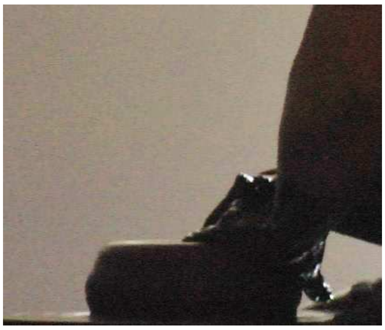

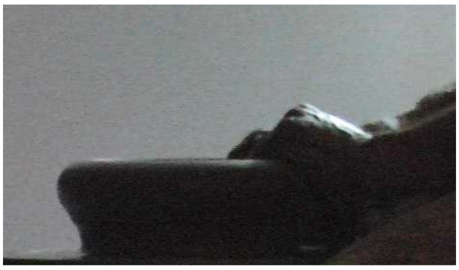

Hand position 52

hand position	small sphere	large sphere	French	Multani
52		x	x	

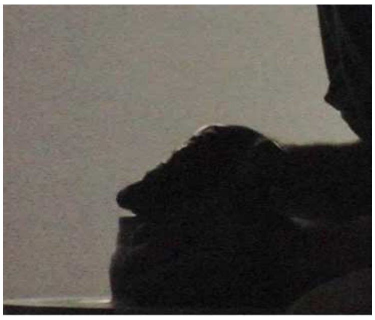

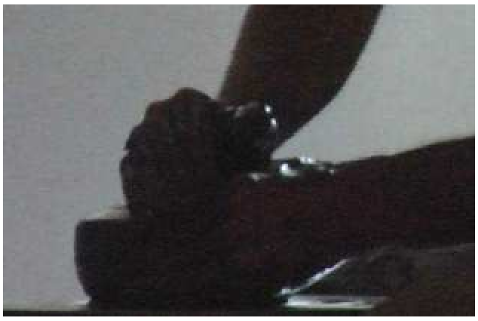

Hand position 53

hand position	small sphere	large sphere	French	Multani
53		x	x	

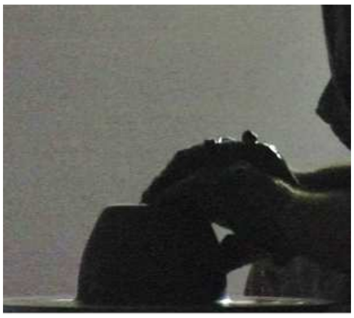

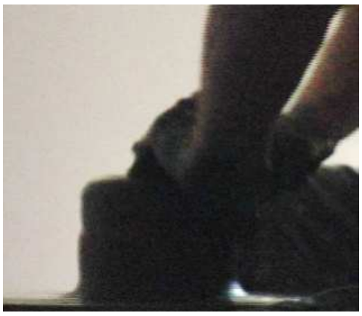

Hand position 54

hand position	small sphere	large sphere	French	Multani
54	x	x	x	

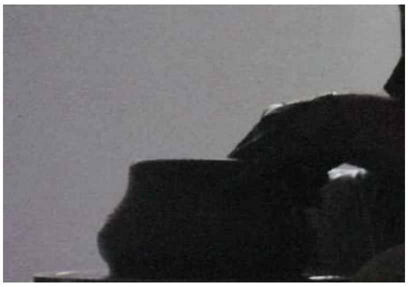 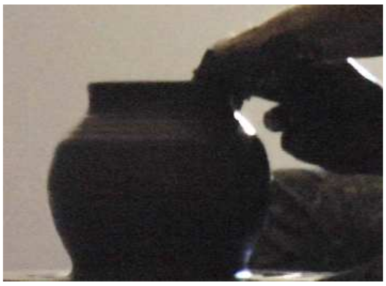 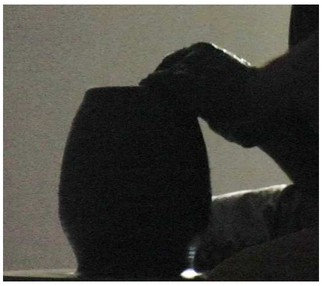

Hand position 55

hand position	small sphere	large sphere	French	Multani
55		x	x	

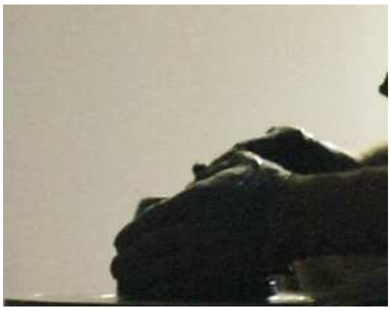 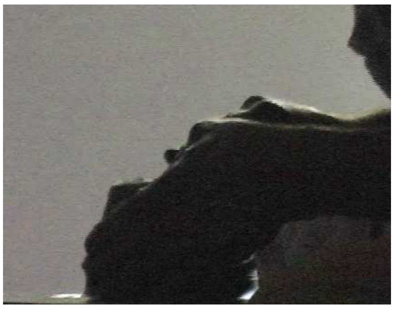

Hand position 56

hand position	small sphere	large sphere	French	Multani
56	x	x	x	

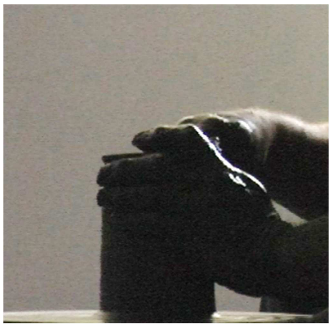 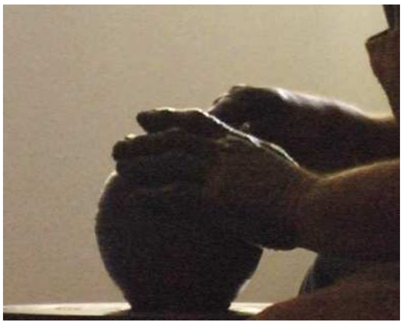 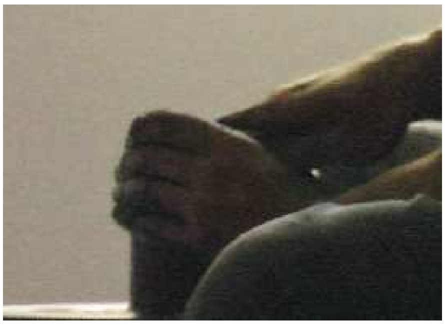

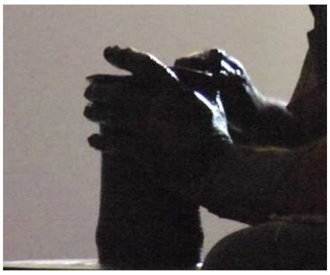

Hand position 57

hand position	small sphere	large sphere	French	Multani
57	x	x	x	

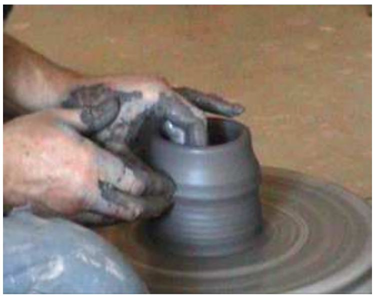

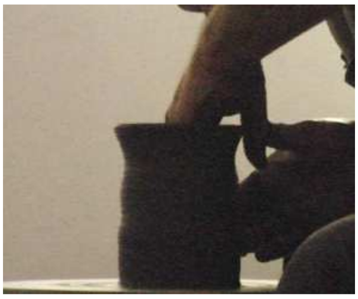

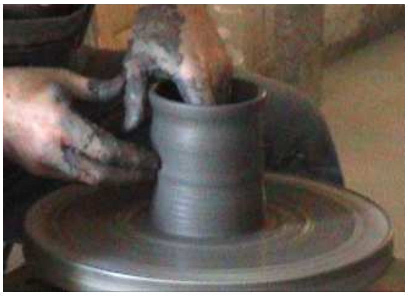

Hand position 58

hand position	small sphere	large sphere	French	Multani
58		x	x	

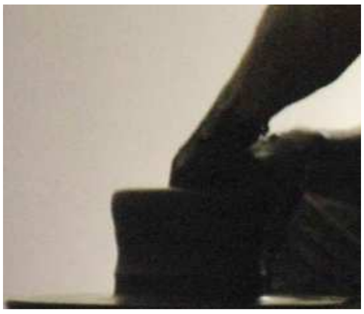

Hand position 59

hand position	small sphere	large sphere	French	Multani
59		x	x	

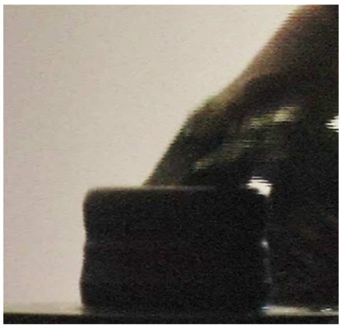 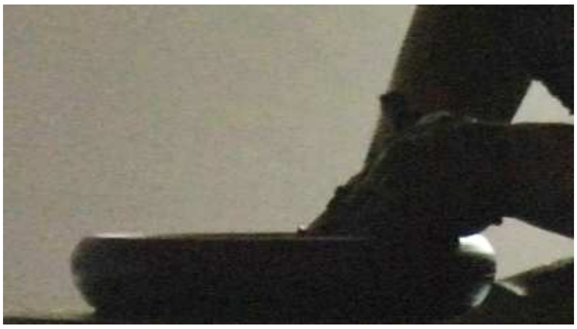

Hand position 60

hand position	small sphere	large sphere	French	Multani
60		x	x	

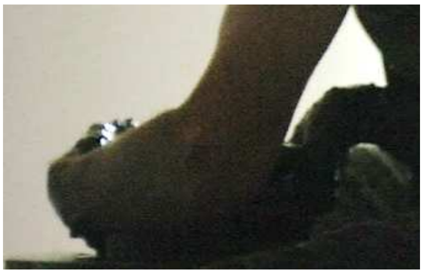 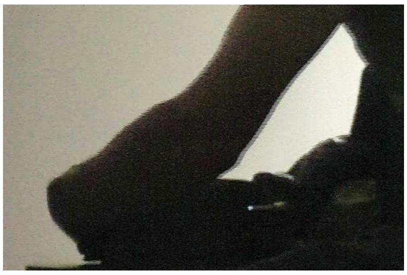

Hand position 61

hand position	small sphere	large sphere	French	Multani
61	x		x	

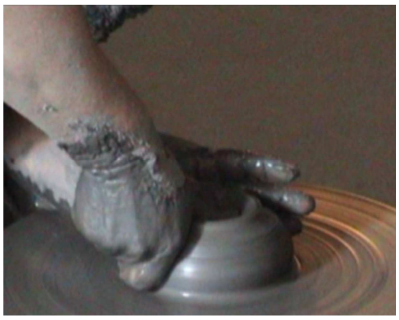 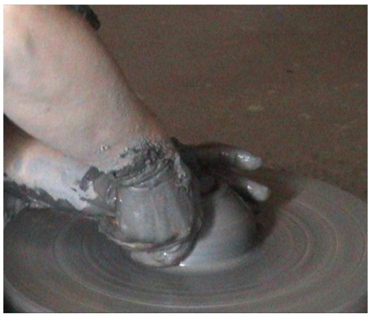

Hand position 62

hand position	small sphere	large sphere	French	Multani
62		x	x	

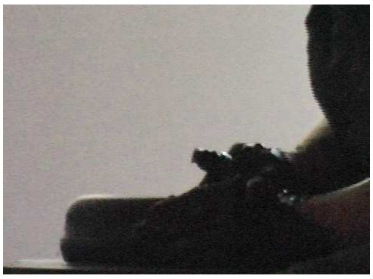
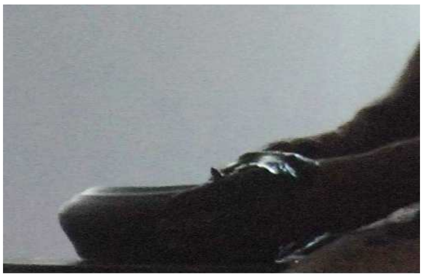

Supplement: Supporting Information S1 — Hand positions repertoire. Photographs of the 62 hand positions identified in the French and Indian Multani groups. (PDF) [file pone.0081614.s001.pdf]
