# Supplementary figures and images for: How Can Ten Fingers Shape a Pot? Evidence for Equivalent Function in Culturally Distinct Motor Skills
Source: PLoS One. 2013 Nov 27;8(11):e81614. doi: 10.1371/journal.pone.0081614 (PMC3842241; doi:10.1371/journal.pone.0081614)

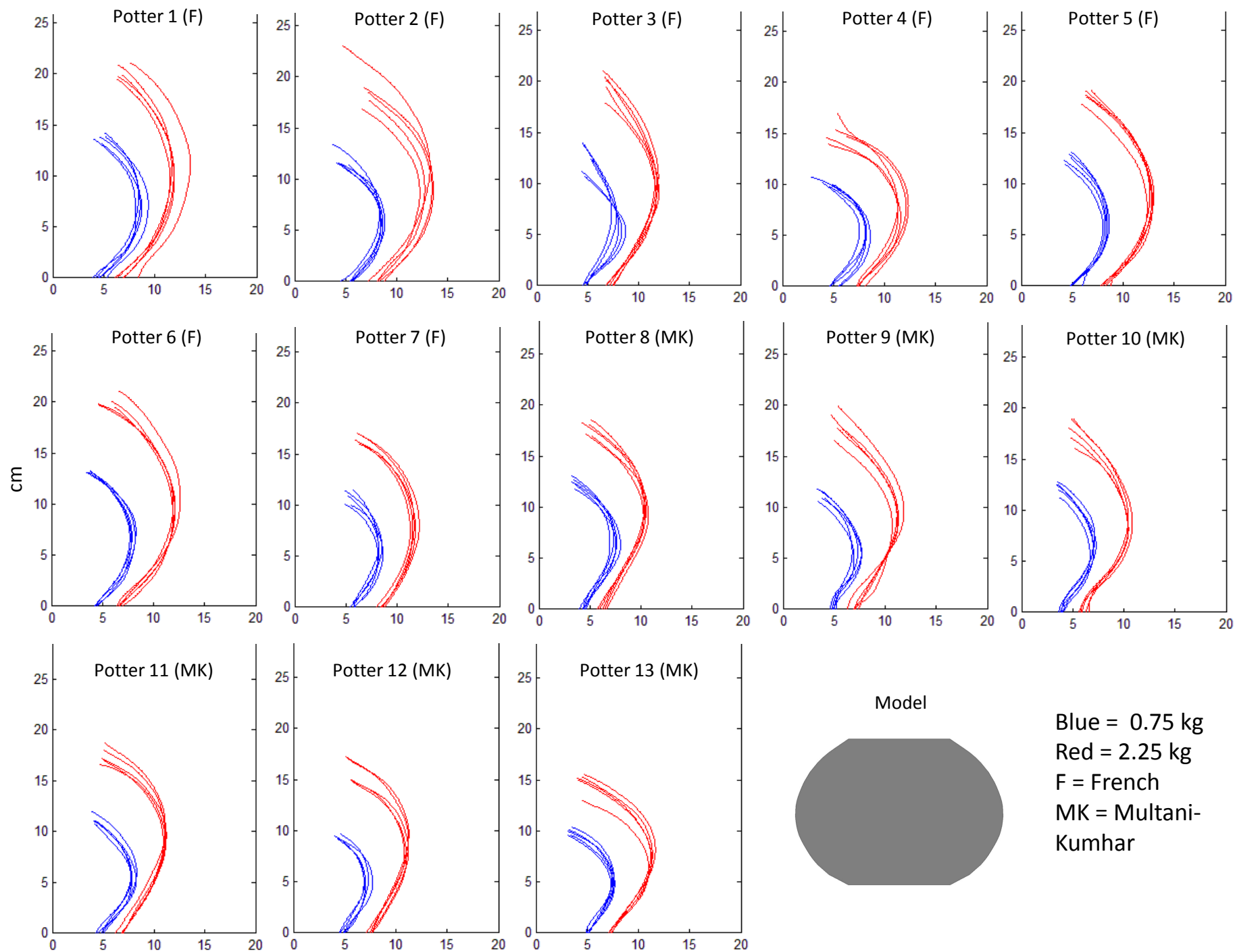

Supplement: Supporting Information S2 — Model shape and 2D cross-sectional profiles of the vessels thrown by individual potters. Blue lines represent the 0.75-kg vessels; red lines represent the 2.25-kg vessels. Potters 1 to 7 constitute the French group and potters 8 to 13 constitute the Indian Multani group. (PDF) [file pone.0081614.s002.pdf]
